# Coronatine-Induced Maize Defense against *Gibberella* Stalk Rot by Activating Antioxidants and Phytohormone Signaling

**DOI:** 10.3390/jof9121155

**Published:** 2023-11-30

**Authors:** Mei Liu, Yiping Sui, Chunxin Yu, Xuncheng Wang, Wei Zhang, Baomin Wang, Jiye Yan, Liusheng Duan

**Affiliations:** 1 Engineering Research Center of Plant Growth Regulator, Ministry of Education & College of Agronomy, China Agricultural University, Beijing 100193, China; 2 Beijing Key Laboratory of Environment Friendly Management on Fruit Diseases and Pests in North China, Institute of Plant Protection, Beijing Academy of Agriculture and Forestry Sciences, Beijing 100097, China; 3 Beijing Key Laboratory of New Technology in Agricultural Application, College of Plant Science and Technology, Beijing University of Agriculture, Beijing 102206, China

**Keywords:** maize, *Gibberella* stalk rot, coronatine, transcriptome, co-expression network, metabolomics, phytohormone signaling, flavonoid biosynthesis

## Abstract

One of the most destructive diseases, *Gibberella* stalk rot (GSR), caused by *Fusarium graminearum*, reduces maize yields significantly. An induced resistance response is a potent and cost-effective plant defense against pathogen attack. The functional counterpart of JAs, coronatine (COR), has attracted a lot of interest recently due to its ability to control plant growth and stimulate secondary metabolism. Although several studies have focused on COR as a plant immune elicitor to improve plant resistance to pathogens, the effectiveness and underlying mechanisms of the suppressive ability against COR to *F*. *graminearum* in maize have been limited. We investigated the potential physiological and molecular mechanisms of COR in modulating maize resistance to *F*. *graminearum*. COR treatment strongly enhanced disease resistance and promoted stomatal closure with H_2_O_2_ accumulation, and 10 μg/mL was confirmed as the best concentration. COR treatment increased defense-related enzyme activity and decreased the malondialdehyde content with enhanced antioxidant enzyme activity. To identify candidate resistance genes and gain insight into the molecular mechanism of GSR resistance associated with COR, we integrated transcriptomic and metabolomic data to systemically explore the defense mechanisms of COR, and multiple hub genes were pinpointed using weighted gene correlation network analysis (WGCNA). We discovered 6 significant modules containing 10 candidate genes: WRKY transcription factor (LOC100279570), calcium-binding protein (LOC100382070), NBR1-like protein (LOC100275089), amino acid permease (LOC100382244), glutathione *S*-transferase (LOC541830), HXXXD-type acyl-transferase (LOC100191608), prolin-rich extensin-like receptor protein kinase (LOC100501564), AP2-like ethylene-responsive transcription factor (LOC100384380), basic leucine zipper (LOC100275351), and glycosyltransferase (LOC606486), which are highly correlated with the jasmonic acid–ethylene signaling pathway and antioxidants. In addition, a core set of metabolites, including alpha-linolenic acid metabolism and flavonoids biosynthesis linked to the hub genes, were identified. Taken together, our research revealed differentially expressed key genes and metabolites, as well as co-expression networks, associated with COR treatment of maize stems after *F. graminearum* infection. In addition, COR-treated maize had higher JA (JA-Ile and Me-JA) levels. We postulated that COR plays a positive role in maize resistance to *F*. *graminearum* by regulating antioxidant levels and the JA signaling pathway, and the flavonoid biosynthesis pathway is also involved in the resistance response against GSR.

## 1. Introduction

Maize (*Zea mays*) is one of the three major food crops used for feed and biofuel materials [1]. The total planting area of maize around the world is 197 million hectares, and the whole yield is 1.15 billion tons of grain [2]. As one of the most economically important crop species worldwide, during their development, maize plants undergo multiple challenges, including pathogenic infections, environmental stress, and insect attacks [3]. Stalk rot, one of the most destructive corn diseases worldwide, is caused by a range of pathogenic fungi and bacteria, including *Colletotrichum graminicola*, *Fusarium graminearum*, *Fusarium verticillioides*, and *Pectobacterium chrysanthemi*, and normally results in substantial yield loss for maize [4]. Moreover, stalk rot retards the extensive application of grain mechanical harvesting techniques. *Gibberella* stalk rot (GSR) is a severe soil-borne disease that has become a significant hazard to maize production. GSR is caused by the fungus *Fusarium graminearum*, which usually overwinters as chlamydospores on crop residue and infects plants through direct penetration of seedling roots or insect- or mechanically caused lesions [5]. It severely reduces the productivity and quality of maize and produces various mycotoxins [6]. *F. graminearum* also causes crown rot and seedling blight, in addition to Fusarium head blight [7]. After pollination, GSR symptoms develop rapidly during mild and humid weather. *F. graminearum*, a hemibiotrophic fungal pathogen, endures a biotrophic phase in its early stage and a necrotrophic phase in its later stage [8]. During infection, *F. graminearum* hyphae emerge intercellularly in the early stage (before 24 h post-inoculation, hpi), intracellularly and intercellularly in the middle stage (36–48 hpi), and grow rapidly in the late stage (after 72 hpi) when the plant cell structure collapses and tissue rots [9]. Multiple immune defense responses are activated to eliminate infective pathogens in plant organs after pathogens invade physical barriers of the plant’s biological structure, such as the cuticle and cell wall [10].

Plants have evolved mechanisms for detecting potential aggressors and coordinating appropriate defensive responses, such as producing toxic substances and lytic enzymes. These responses are regulated by phytohormones, with jasmonic acid (JA), salicylic acid (SA), and ethylene serving as the primary regulators. In general, JA coordinates responses effectively against necrotrophic pathogens and chewing insects, whereas SA targets primarily biotrophic pathogens [11]. Some research has focused on plants that respond to *F. graminearum*, such as wheat and corn. To limit *F. graminearum* infection, plants use defense-related hormones, reactive oxygen species (ROS), pathogenesis-related (PR) proteins, and cellular detoxification-related proteins [12]. GSR resistance is a quantitative trait; only a small number of QTLs or genes have been identified or cloned in maize [13]. Thus, due to the soil-borne infection pattern of *F. graminearum* and the fact that only a small number of maize alleles confer resistance to GSR, it is difficult to control with fungicides and other chemical treatment approaches. Considering the significance of eco-friendly and sustainable agricultural practices, many studies on stalk rot resistance have been conducted to further control this disease [9]. GSR control strategies have shifted toward the application of plant immune elicitors rather than harmful chemical pesticides.

Coronatine (COR) is a phytotoxin generated by *Pseudomonas syringae*. This compound has recently received much attention due to its potential as a plant growth regulator and elicitor of plant secondary metabolism [14]. COR appears to share structural and functional similarities with JA and related signaling compounds, including methyl jasmonate (MeJA) and 12-oxo-phytodienoic acid (12-OPDA), the C_18_ precursor of JA and MeJA. JA and 12-OPDA regulate many plant functions, particularly the activation of secondary metabolism, by acting as signaling compounds in plant defensive responses against several stressors [15]. According to Svoboda (2010), the phytohormones JA and its isoleucine-conjugated form (JA–Ile) play a significant role in the defense responses of plants to various external plant invaders. It is well known that JAs regulate pathogen defense via the homeostasis of active JAs and the COI-JAZ-MYC function module [16]. The multiple roles of JAs in maize are best illustrated in the study of the *opr7opr8* double mutant, which is deficient in JA biosynthesis and susceptible to the soil-borne pathogens *Pythium* spp. and *Fusarium* verticillioides [17] but resistant to the anthracnose leaf blight, which is caused by *Colletotrichum graminicola* [18]. In a pair of wheat lines with differing levels of resistance to Fusarium head blight (FHB), it was discovered that several genes of JA signaling pathways were upregulated in the resistant line [19]. A recent study revealed that exogenous treatment of maize with 12-OPDA increased resistance to maize anthracnose leaf blight caused by *Colletotrichum graminicola* [20]. Furthermore, exogenous treatment of maize with MeJA significantly enhanced resistance to GSR [21].

COR resembles the conjugate JA–Ile, so its mode of action may be similar. COR has been used to design structural analogs at the level of synthetic chemistry that could be applied in agriculture as stimulators of plant defense mechanisms. Exogenously applied COR enhances cabbage seedlings’ resistance to *Xanthomonas campestris* pv. *campestris*, which might be related to variations in antioxidant enzyme activity, the O^2-^generation rate, and malondialdehyde (MDA) content [22]. Additionally, COR enhances mulberry seedlings’ resistance to *P. syringae* pv. *mori* by producing signal molecules to activate signaling pathways and promote defense-related metabolism [23]. Due to the rigid cis-orientation of its bicyclic skeleton, COR’s chemical structure is significantly more stable [24]. This may explain the higher levels of secondary metabolism induction observed in plant/cell cultures treated with COR compared to those treated with ‘natural’ JAs [14].

COR is environmentally benign and safe for animals and humans, and exogenous COR treatment may be an effective strategy to protect maize from pathogen infection. To study the possible mechanism of COR’s influence on maize resistance to GSR, antioxidant and defense enzyme activities, H_2_O_2_ and MDA content, stomatal density and aperture, phytohormone levels, and the relative expressions of various defense-related signaling pathway genes were detected. To explore the genome-wide transcriptional and metabolomic alterations of maize infected with *F. graminearum* and treated by COR, integrated studies employing RNA-seq and weighted gene co-expression network analysis (WGCNA) in conjunction with metabolomics were deployed. Using the Kyoto Encyclopedia of Genes and Genomes (KEGG), Gene Ontology (GO) clustering, the Weighted Gene Co-expression Network Analysis (WGCNA), multiple differentially expressed genes (DEGs), differentially accumulated metabolites (DAMs), and co-expression networks regulated by hub genes that are probably associated with GSR resistance were identified. 

Therefore, in this study, we explored the phenotypic, transcriptomic, and metabolomic changes following treatment of COR on maize seedlings, and we also examined the effectiveness of COR treatment on alleviating the fungal disease *Gibberella* stalk rot caused by *Fusarium graminearum*. Our study aimed to explore the key metabolic pathways and regulatory factors and elucidate the possible underlying mode mechanism of COR-induced *F. graminearum* resistance in maize. This study provides new insights into the mechanism related to maize infected by *F. graminearum’s* response to COR, and the results can provide a theoretical basis for green prevention and control of GSR.

## 2. Materials and Methods

### 2.1. Plant Material and Treatments

The maize-susceptible variety “Zhengdan 958” was grown in greenhouses at the Engineering Research Center of Plant Growth Regulator, College of Agronomy, China Agricultural University, Beijing 100193, China. The maize seeds were sown in the soil in long pots made with PVC cylinders. The pots were maintained on plant growth platforms for two weeks, with a light intensity of 600 Lux and a relative humidity of approximately 75%. Selected seedlings of similar size were transferred to a culture chamber for the phenotypic assay. The seedlings were then sprayed with 1, 10, or 50 μg/mL of COR dissolved in 0.1% acetone, and 10–14 days post-infection (dpi) stems were harvested or used for the GSR phenotype assay. For RNA-seq analysis or other investigations, stem tissues were collected at various times (e.g., 0, 6, 12, 24, 48, 72, and 120 hpi), promptly frozen in liquid N_2_, and stored at −80 °C. The control plants received an identical concentration of 0.1% acetone. Each treatment per time point consists of at least ten seedlings for transcriptomic, ten for metabolomic, and ten for GSR phenotyping. The experiments were conducted in at least three biological replicates, with constant results obtained.

### 2.2. Disease Phenotype Investigation and Maize Growth

The GSR assay was performed as previously described by Sun et al., (2018), with minor modifications [25]. Then, 48 h after treatment with 1, 10, or 50 μg/mL COR, the stems were infected with a spore suspension of *F. graminearum* (1.0 × 10^6^ spores in 0.001% Tween-20). GSR symptoms were evaluated at 10 dpi. After 72 h of *F. graminearum* infection of maize stems, 10 sections were stained with a GMS stain kit (Baso Diagnostics Inc., Zhuhai, China) for each treatment. We also stained the sections with a PAS stain kit (Baso Diagnostics Inc., Zhuhai, China). After cleansing and dehydrating, the slides were filled with a neutral resin for microscopic examination [26]. Maize stems inoculated with *F. graminearum* for 72 h were collected, fixed, and used for scanning electronic microscopy observations, with 10 sections for each treatment. According to Jin et al. [27], diseased sections of stem segments measuring approximately 5 mm in length were fixed, subsequently treated, and observed. In addition, the height, stem diameter, leaf length, leaf width, fresh weight, dried weight, and SPAD (Soil and Plant Analyzer Development) of 10 seedlings from each of the three treatments were measured.

### 2.3. Assay of Activities of Antioxidant Enzymes, Defense Enzymes, and MDA Content

The activities of antioxidant enzymes, including catalase (CAT), superoxide dismutase (SOD), peroxidase (POD), and defense enzymes, including polyphenol oxidase (PPO), phenylalanine ammonia-lyase (PAL), and lipoxygenase (LOX), as well as the content of malondialdehyde (MDA), were estimated in maize stems treated with 1 and 10 μg/mL COR at 0, 24, 48, 72, and 120 hpi according to the method described in Zhang et al. [28]. For analysis of the activities of SOD, POD, and CAT, 0.5 g of frozen stem sample was homogenized with 5 mL of cold 100 mM PBS (pH 7.0). All extraction procedures were conducted at 4 °C. The homogenate was centrifuged at 12,000× *g* for 15 min at 4 °C, and the supernatants were collected., For analysis of the activities of PPO, PAL, and LOX, 0.5 g of frozen stem sample was extracted with 5 mL of extraction buffer (100 mM, pH 6.8 (PBS) for PPO; 0.2 mM pH 8.8 boric acid buffer, containing 10% (*w*/*v*) polyvinylpyrrolidone and 1 mM EDTA for PAL; 100 mM pH 5.2 acetic acid buffer for LOX) and centrifuged at 12,000× *g* for 20 min at 4 °C. For the assay of melatonin content, 1 g of stem sample was transferred to 5 mL of extraction mixture (acetone:methanol:water = 89:10:1) and the homogenate was centrifuged at 12,000× *g* for 20 min at 4 °C. All measurements were performed in triplicate, with samples collected from three biological replicates.

### 2.4. Measurement of Stomatal Density and Aperture

The maize stems of 14-day-old seedlings treated with 10 μg/mL COR and under normal conditions at 24 hpi were coated and fixed with nail polish, and the stomatal density was observed through a microscope (Imager Z2, Zeiss, Jena, Germany). At room temperature, samples were fixed in 2.5% glutaraldehyde, dehydrated through an ethanol series (50, 70, 90, and 100%), and dried with a critical drying oven. The stomatal aperture on the stem was observed to be about a third of its length from the inoculated dot using confocal scanning electron microscopy (LSM880, Zeiss, Jena, Germany). For each biological replicate, fifty stomata from ten stems were measured [29].

### 2.5. Assay of H_2_O_2_ Accumulation

An H_2_O_2_ assay kit (Nanjing Jiancheng Bioengineering Institute, Nanjing, China) was used to measure the H_2_O_2_ content. 0.5 g of frozen stem sample was homogenized with 5 mL of cold 100 mM PBS (pH 7.0). All extraction procedures were conducted at 4 °C. The homogenate was centrifuged at 12,000× *g* for 15 min at 4 °C, and the supernatants were collected. All measurements were conducted in triplicate, using samples from three biological replicates. The 2′,7′-dichlorodihydrofluorescein diacetate (H_2_DCF-DA) staining assay was performed, as previously described [30]. Briefly, maize stems treated with 10 μg/mL COR and under normal conditions at 24 hpi were vacuum-infiltrated in 0.01% Tween-20 for 5 min and then incubated in 2% (*w*/*v*) cellulase at 37 °C for 3 h to peel off the epidermal layers. Samples were transferred to a staining buffer (50 mM H_2_DCF-DA, 10 mM Tris-HCl, 50 mM KCl, pH 7.2) for 20 min. The samples were washed with ddH_2_O three times. Using a confocal laser scanning microscope (ZEISS, LSM710, Jena, Germany) with excitation at 488 nm and emission at 546 nm, the fluorescence of the guard cells was observed on the stem about a third of the way from the inoculation site.

### 2.6. Quantification of Phytohormones

The biologically active metabolites MeJA, JA-Ile, OPDA, SA, SAG, and ACC related to the endogenous stress-related hormones JA, SA, and ET were extracted at different time points (0, 24, and 72 h) from maize stems detected by MetWare (MetWare Biotechnology Co., Ltd., Wuhan, China). Briefly, frozen maize stems were ground into a fine powder using a mixer mill (MM 400, Retsch, Haan, Germany) for 1.5 min at 30 Hz. Then, 50 mg of the powdered maize stems were extracted with methanol/water/formic acid (15:4:1, *V*/*V*/*V*) at 4 °C overnight, and ultrasound-assisted extraction was conducted at room temperature for 30 min with vortexing (15 s), followed by centrifugation at 14,000 rpm for 10 min. A total of 1000 μL of supernatant was collected, evaporated to dryness with nitrogen gas at room temperature, reconstituted in 100 μL of 80% (*v*/*v*) methanol, and diluted to 800 μL with water. Extracts were then passed through an SPE cartridge (CNWBOND carbon-GCB, 250 mg, 3 mL; ANPEL, Shanghai, China) and evaporated to dryness using nitrogen gas at room temperature. The extracts were absorbed using a CNWBOND CarbonGCB SPE Cartridge (250 mg, 3 mL) and filtered using an SCAA-104 Cartridge (0.22 mm pore size) (ANPEL, Shanghai, China). As previously described [31], LC-MS analysis was conducted using a liquid chromatography-electrospray ionization-tandem mass spectrometry (LC-ESI-MS/MS) system (high-performance liquid chromatography (HPLC): Shim-pack UFLC Shimadzu CBM30A system, Kyoto, Japan; MS, Applied Biosystems 4500 Q TRAP). For each time point, three replicates were analyzed.

### 2.7. RNA-Seq Transcriptome Analysis and qRT-PCR Validation

Using a TIANGEN reagent (Beijing, China), total RNA was extracted from the stem samples at the specified time points (24 and 72 hpi) following the manufacturer’s instructions, and index codes were added to the attribute sequences of each sample. Using the DNBSEQ platform, libraries were compiled and generated. The RNA-seq library was constructed using Illumina HiSeq 2000 (Illumina Inc., San Diego, CA, USA) by the Berry Genomics company (Beijing, China). Paired-end RNA sequence reads were generated, cleaned, and aligned to the B73 reference genome (RefGen_V4) using Hisat2 [21]. The relative expression of transcripts was calculated based on the number of fragments per kilobase of exons per million mapped reads (FPKM) by using HTSeq 0.12.4 [32]. The DEGs were selected with an adjusted *p* value (*p.adj*) < 0.05, log2Foldchange > 1 for upregulated genes, and log2Foldchange < 1 for downregulated genes relative to the control. The Kyoto Encyclopedia of Genes and Genomes (KEGG) pathway analysis of differentially expressed genes was mapped using KEGG Orthology databases [33].

cDNA was synthesized according to the manufacturer’s instructions using Oligo d (T) primer and M-MLV reverse transcriptase (Takara, Japan). Quantitative RT-PCR analysis was performed using the SYBR^®^ Premix Ex Taq (Takara, Japan) in an Applied Biosystems 7500 Fast Real-Time PCR System (Applied Biosystems, Waltham, MA, USA). Initial denaturation was at 95 °C for 30 s, followed by 40 cycles of 95 °C for 5 s and 60 °C for 34 s. Each quantitative RT-PCR reaction included three technical replicates per sample. The transcript level of the ZmUbiquitin gene (GRMZM2G409726) was used for internal normalization. Each experiment included at least three biological replicates. Primers for qRT-PCR are listed in Supplementary Table S1.

### 2.8. WGCNA for Identification of Key Candidate Genes Involved in Maize Defense Associated with COR

WGCNA was performed in R (4.1.2) using the default parameters to simplify genes into expressed modules and identify defense-related hub genes [34]. Normalized FPKM values were used to construct an adjacency matrix. The plant antioxidant and defense enzymes, used as phenotypic data, were imported into the WGCNA package, and the correlation between antioxidants and gene modules was calculated using the default settings. The adjacency matrix was converted into a topological overlap matrix (TOM) using the WGCNA program. After constructing a network, transcripts with identical expression patterns were grouped into modules, and their eigengenes were calculated for these modules. Each module’s genes were exported using the default Cytoscape export parameters [35].

### 2.9. Detection and Analysis of Metabolites

The maize stems treated with 10 μg/mL COR and under normal conditions at 24 h infestation were collected at 24 and 72 hpi. MetWare Company (Wuhan, China) provided sample preparation and metabolomic analysis according to the manufacturer’s protocols, with some modifications. The MetWare Database (Wuhan MetWare Bio-technology Co., Ltd., Wuhan, China) and publicly available metabolites were used to identify the metabolites. The supervised multivariate method orthogonal projections to latent structures discriminant analysis (OPLS-DA) was used to identify differentially accumulated metabolites (DAMs) between stems sprayed with 10 g/mL COR and stems infected with *F. graminearum*. The relative significance of each metabolite to the OPLS-DA model was analyzed using the variable importance in projection (VIP) metric. For group comparisons, metabolites with VIP ≥ 1.0 and fold change ≥ 2 or fold change ≤ 0.5 were considered DAMs [36]. Annotated metabolites were then mapped to the KEGG Pathway database using the KEGG Compound database6. Then, metabolite set enrichment analysis (MSEA) was applied to the pathways with substantially regulated metabolites, and hypergeometric test *p* values were used to determine the significance of DAMs.

## 3. Results

### 3.1. Disease Phenotype Investigation and Maize Growth

**Figure 1:**
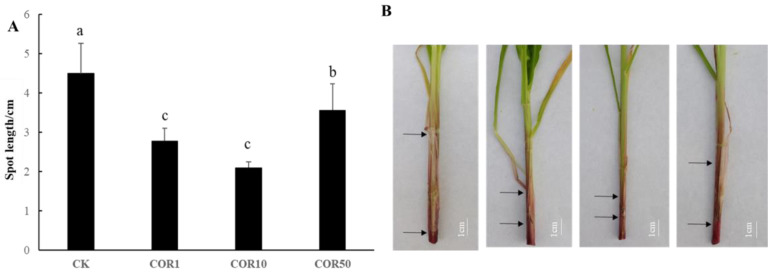
Exogenous COR enhances maize disease resistance against *Fusarium graminearum*. (**A**) GSR phenotype of Zhengdan 958 sprayed with COR and CK, infected with *F. graminearum*. (**B**) Quantification of the disease length on the plant stems from A. Mock represents the treatment with the same amount of 0.1% acetone. Significant differences between treatments are denoted by the use of lowercase letters of a different case above each bar (*p* < 0.05). Treatments include the untreated control (CK), treatment with 1 μg/mL COR (COR1), treatment with 10 μg/mL COR (COR10), and treatment with 50 μg/mL COR (COR50). The black arrow delineates the area of disease.

To determine whether COR contributes to maize resistance to GSR, 2-week-old “Zhengdan958” seedlings were sprayed with COR for 48 h and then inoculated with an *F. graminearum* spore suspension at a concentration of 1.0 × 10^6^ spores/mL. COR application at different concentrations (1, 10, and 50 μg/mL) significantly reduced the lesion length in maize stems compared with the control. In addition, the lesion length was not significantly different between the 1 and 10 μg/mL COR treatment groups (Figure 1).

**Figure 2:**
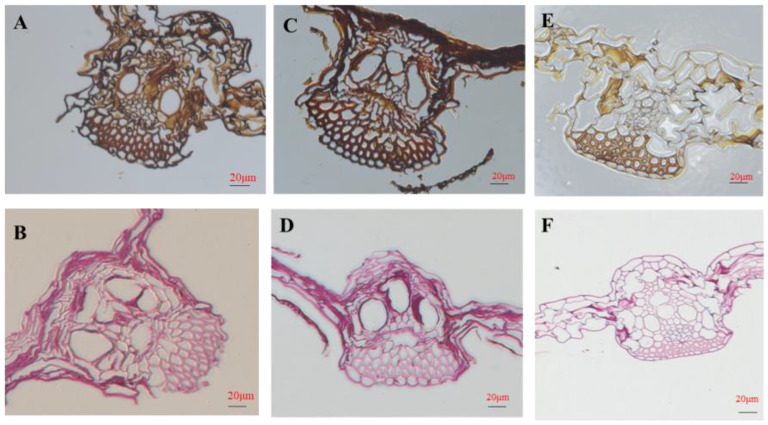
GMS and PAS staining of diseased maize stem tissues observable by their black-brown and magenta colors, respectively. (**A**) GMS staining of CK-treated maize stems. (**B**) PAS staining of CK-treated maize stems. (**C**) GMS staining of COR1-treated maize stems. (**D**) PAS staining of COR1-treated maize stems. (**E**) GMS staining of COR10-treated maize stems. (**F**) PAS staining of COR10-treated maize stems. Treatments include the administration of CK, 1 μg/mL COR (COR1), and 10 μg/mL COR (COR10).

After *F. graminearum* infection of the maize stems for 72 h, images of fungal hyphae were visualized and compared using GMS and PAS staining (Figure 2). The presence of black-brown fungal hyphae was indicative of a positive diagnosis of GMS. The presence of magenta fungal hyphae confirmed a positive diagnosis of PAS. The outcomes were identical when comparing the sensitivity of GMS staining and PAS staining for all fungal infections. In terms of the dyeing area and strength, the effect of 10 μg/mL COR-treated stems was weaker than that of the control stems. To determine how *F. graminearum* hyphae invaded maize stems, the regions around the inoculation site were collected at 72 hpi and subjected to scanning electron microscopy. Following inoculation of *F. graminearum* without COR treatment, the surface of diseased regions and the stem segments immediately adjacent to lesions (5 mm above the inoculation site) were densely covered with fungal hyphae. When the maize stems were treated with 10 μg/mL COR, there were fewer densely packed fungal hyphae on the surface of diseased and adjacent regions (Supplementary Figure S1). To investigate the impacts of COR on maize growth, COR application at the seedling stage was found to significantly decrease the stem diameter. In addition, 1 μg/mL COR treatment significantly increased the leaf length and significantly decreased the leaf width, and 10 μg/mL COR treatment significantly decreased plant height and leaf width. The application of 50 μg/mL COR significantly decreased plant height, leaf length, leaf width, fresh weight, and dry weight. There was no significant difference between treatments for SPAD (Table 1). 

### 3.2. Effects of COR on MDA Accumulation, Activities of Antioxidants and Defense-Related Enzymes

**Figure 3:**
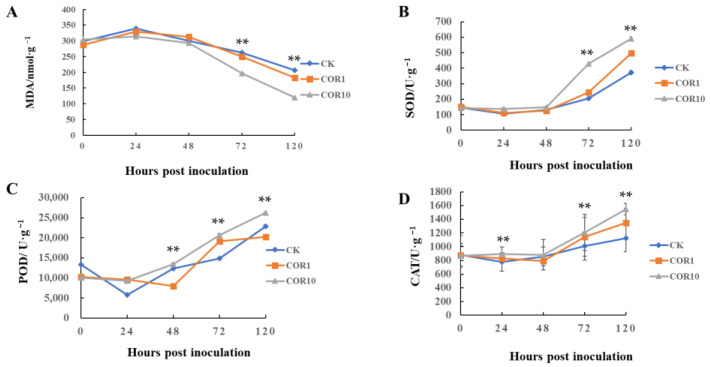
Accumulation of MDA and the activities of antioxidant enzymes. (**A**) MDA content. (**B**) SOD activity. (**C**) POD activity. (**D**) CAT activity. Significant differences between the control group and the 1 μg/mL and 10 μg/mL COR-treated groups were compared using Duncan’s multiple range test. Significant differences at *p* < 0.01 were marked as double (**) the control group. The data (mean ± SD) were calculated using three replicates.

To better understand the mechanism of COR-induced resistance against *F. graminearum*, the MDA content and activities of antioxidants and defense-related enzymes were determined at 0, 24, 48, 72, and 120 h after 1 and 10 μg/mL COR treatment. Although the MDA content of control, 1 and 10 μg/mL COR−treated stems varied similarly, the content of COR-treated stems decreased significantly compared to the control (Figure 3). 

**Figure 4:**
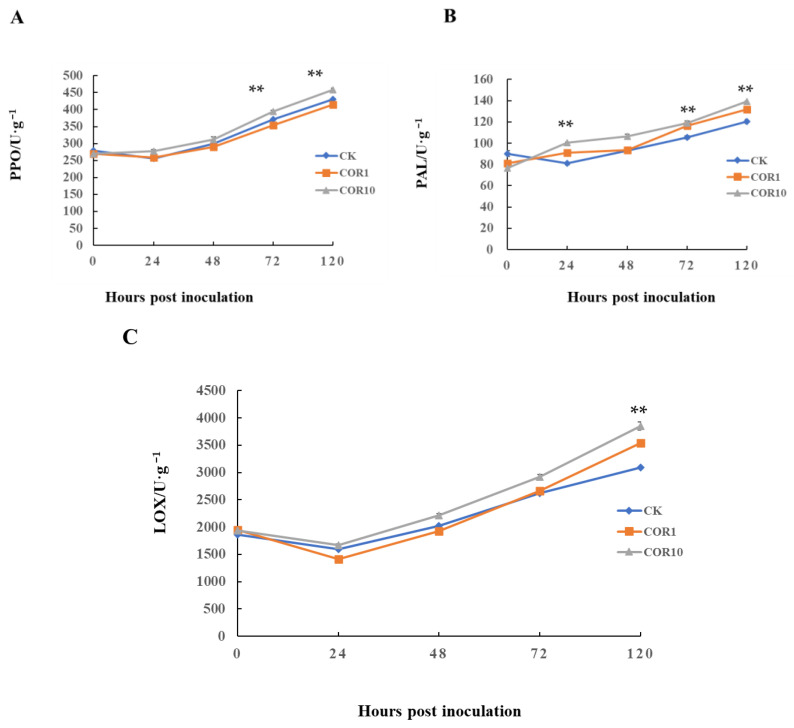
Activities of defense enzymes. (**A**) PPO activity, (**B**) PAL activity, and (**C**) LOX activity. Significant differences between the control group and the COR-treated group were compared using Duncan’s multiple range test. Significant differences at *p* < 0.01 were marked as double (**) the control group. The data (mean ± SD) were calculated using three replicates.

Throughout the majority of the observation period, the activities of antioxidant enzymes (POD, SOD, and CAT) in COR-treated stems were significantly higher than in control stems. SOD and POD activity in COR-treated and control stems rose markedly at 48 h and peaked at 120 h in COR-treated stems, greater than in control stems. In addition, PPO, PAL, and LOX activity in 10 μg/mL COR−treated stems were always higher than that of 1 μg/mL COR-treated and control stems. The PPO activity fluctuated similarly in control and 1 μg/mL COR-treated stems, while the PPO activity in 10 μg/mL COR−treated stems sharply increased at 24 h and was at a maximum until 120 h (Figure 4). The results indicated that 10 μg/mL COR treatment more obviously enhanced disease resistance against *F. graminearum* by increasing the activities of antioxidants and defense-related enzymes.

### 3.3. COR Regulates Stomatal Closure

The effects of COR were investigated on the stomatal density, aperture size, and shape of maize using a light microscope and an electron scanning microscope. Although the stomatal density of the 10 μg/mL COR-treated stems and control stems was similar, the 10 μg/mL COR-treated stems significantly decreased the average stomatal length and width; as a result, COR significantly decreased the average stomatal area. In addition, COR dramatically changed the stomatal aperture area with a substantial decrease in the average stomatal aperture length and width (Table 2).

### 3.4. Effects of COR on H_2_O_2_ Accumulation

**Figure 5:**
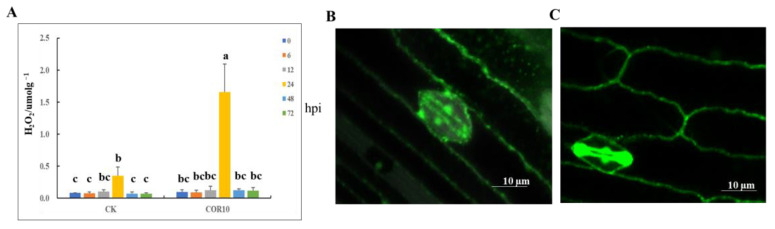
COR positively regulates stomatal closure by inducing the accumulation of hydrogen peroxide (H_2_O_2_). (**A**) H_2_O_2_ content. (**B**) Images of H_2_O_2_−based fluorescence signal in guard cells of maize stem under normal conditions. (**C**) Images of H_2_O_2_−based fluorescence signal in guard cells of maize stem treated with 10 μg/mL COR. Bars, 5 μm. The data are the averages of three independent tests. Different lowercase letters above each bar represent statistically significant differences between treatments (*p <* 0.05). Treatments include the untreated control (CK) and treatment with 10 μg/mL COR (COR10).

The H_2_O_2_ concentration in the control and 10 μg/mL COR−treated stems varied similarly and peaked at 24 h, but the H_2_O_2_ content in the COR-treated stems was significantly higher than that in the control and dropped sharply over the following period. This suggests that COR increased the activities of antioxidant enzymes to scavenge reactive oxygen species (ROS), which is in accordance with the finding that COR increased antioxidative processes and oxidation repair to reduce GSR in maize stems. The accumulation of ROS, such as H_2_O_2_, in guard cells is an early hallmark of stomatal movement. Therefore, we also observed H_2_O_2_ accumulation in the guard cells using an H_2_O_2_-selective 2′,7′-dichlorofluorescein diacetate (H_2_DCF-DA) staining assay under normal conditions and COR treatment with *F. graminearum* infection at 24 h using a confocal laser scanning microscope. Consistent with the changes in the stomatal aperture, the H_2_O_2_−based fluorescence in guard cells, indicating the H_2_O_2_ content, was less intense in the stems of the control plants, whereas it was more intense in the stems of the 10 μg/mL COR−treated plants. Under *F. graminearum* infection, these data support the conclusion that COR induces maize defense by promoting stomatal closure and H_2_O_2_ accumulation in guard cells (Figure 5).

### 3.5. COR Enhanced the Jasmonate (JA-Ile and Me-JA) Concentration after Fusarium graminearum Infection

**Figure 6:**
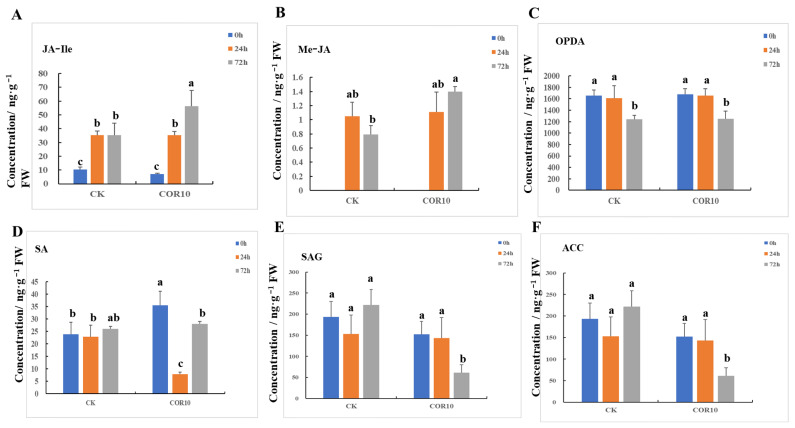
Phytohormone concentration in maize plants. (**A**) JA-Ile concentration. (**B**) Me-JA concentration. (**C**) OPDA concentration. (**D**) SA concentration. (**E**) SAG concentration. (**F**) ACC concentration. Different lowercase letters above each bar represent statistically significant differences between treatments (*p <* 0.05). Treatments include the untreated control (CK) and treatment with 10 μg/mL COR (COR10).

To determine alterations in CORrelated maize stem phytohormones upon infection with *F. graminearum*, we quantified JA-Ile, Me-JA, OPDA, SA, SAG, and ACC. A significant increase in the contents of JA-Ile and Me-JA was recorded in COR-treated maize stems at 72 hpi, while the contents of SAG and ACC were significantly decreased (Figure 6).

### 3.6. Transcriptome Responses Triggered by COR and Validation by qRT-PCR

**Figure 7:**
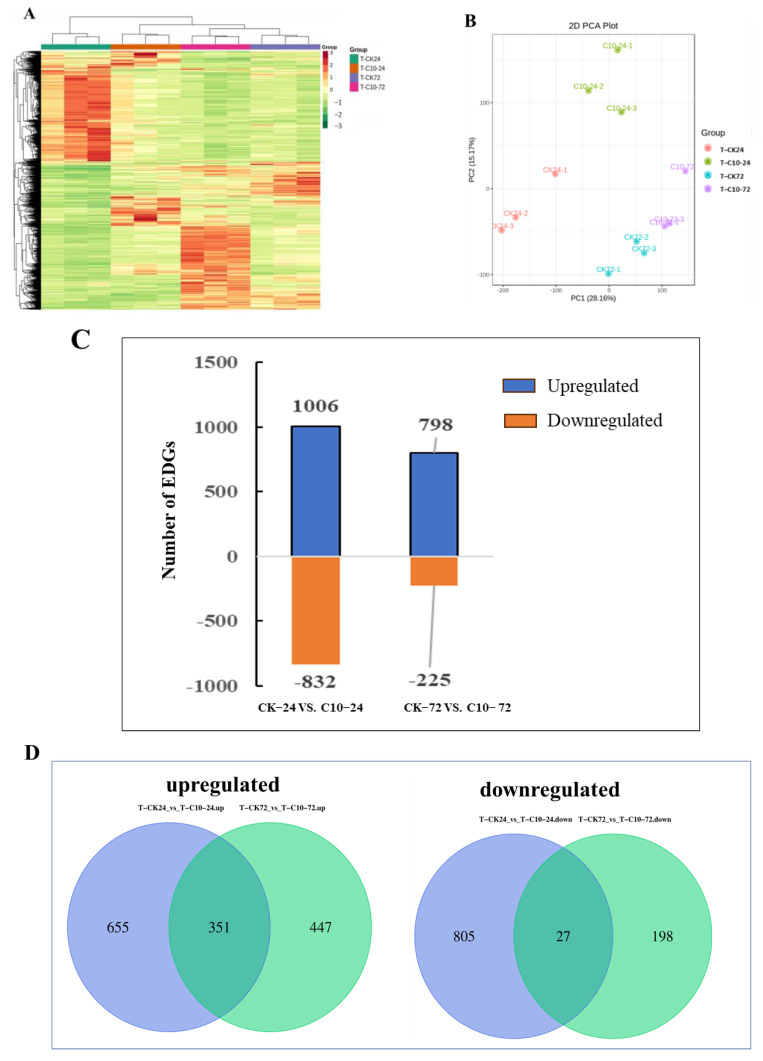
Transcriptome response of maize treated with 10 μg/mL COR and *Fusarium graminearum*. (**A**) Hierarchical cluster dendrogram of differentially expressed genes; the color difference represents high (red) and low (green) expression. (**B**) PCA plot illustrating transcriptome data variability. (**C**) Number of differentially expressed genes (DEGs) that are up−and downregulated in maize stems. (**D**) Venn diagrams illustrating the unique and shared DEGs that were up− or downregulated.

For transcriptome profiling, two treatments (CK and C10) in three replicates at 24 and 72 hpi with *F. graminearum* infection were used to construct 12 cDNA libraries. A total of 86.38 Gb of clean data were obtained, and the clean data of each sample reached 6 Gb. The Q30 base percentage exceeded 91%. The efficiency ranged between 85.23 and 88.75% (Supplementary Table S2) when clean sequences were aligned with a designated reference genome (ZmB73_RefGen_v4). Based on the comparison results, differentially expressed genes (DEGs) were identified based on their expression levels with |log2 (fold-change)|> 1 and an adjusted *p* value < 0.05 in each pairwise comparison. DEGs in all samples were hierarchically clustered relative to the control, and across all time points, the abundance of upregulated genes was greater than that of downregulated genes (Supplementary Tables S3 and S4). The difference in color indicates high (red) and low (green) expressions (Figure 7A). The first two principal components (PCs) of the principal component analysis (PCA) of all detected genes explained 43.33% of the total variation (Figure 7B). The clustering of biological samples from the same time point suggests that each treatment is repeatable. The dispersed clustering of samples from various time points indicates that the time that the stems had been subjected to *F. graminearum* infection had varying effects on maize transcriptome profiles. In total, 1006 (798) DEGs were upregulated, and 832 (225) DEGs were downregulated in the samples treated with COR for 24 and 72 h, respectively (Figure 7C). Among these DEGs, 351 were upregulated at both time points, and 27 were shared among the downregulated genes (Figure 7D).

Using Blast2GO (GOseq R package: http://www.geneontology.org, accessed on 10 April 2023), the DEGs were classified into three GO categories: Biological process (BP), cellular component (CC), and molecular function (MF). In a comparison of CK_24h vs. C10_24h, 1266 DEGs were enriched into 2675 GO terms, and the top 5 (*q* value ≤ 0.05) terms that were significantly enriched were DNA packaging complex (GO: 0044815; *q* value = 4.8 × 10^−35^), nucleosome (GO: 0000786; *q* value = 1.4 × 10^−30^), mitotic cell cycle process (GO: 1903047; *q* value = 1.8 × 10^−22^), protein–DNA complex (GO: 0032993; *q* value = 2.4 × 10^−19^), and DNA packaging (GO: 0006323; *q* value = 9.7 × 10^−18^) (Supplementary Table S5, Supplementary Figure S2). In a comparison of CK_72h vs. C10_72h, 694 DEGs were enriched into 1974 GO terms, and the top 10 (*q* value ≤ 0.05) terms that were significantly enriched were response to wounding (GO: 0009611; *q* value = 1.5 × 10^−11^), hormone metabolic process (GO: 0042445; *q* value = 1.2 × 10^−9^), terpenoid metabolic process (GO: 0006721; *q* value = 4.6 × 10^−9^), monooxygenase activity (GO: 0016491; *q* value = 3.6 × 10^−8^), response to chitin (GO: 0010200; *q* value = 3.6 × 10^−8^), terpene synthase activity (GO: 0016491; *q* value = 3.6 × 10^−8^), isoprenoid metabolic process (GO: 0006720; *q* value = 5.7 × 10^−7^), response to jasmonic acid (GO: 0009753; *q* value = 5.7 × 10^−7^, iron ion binding (GO: 0006720; *q* value = 7.3 × 10^−7^), negative regulation of gibberellic acid mediated signaling pathway (GO: 0009938, *q* value = 1.5× 10^−6^), carbon-oxygen lyase activity, acting on phosphates (GO: 0016838, *q* value = 1.7× 10^−6^), and defense response to fungus (GO: 0050832, *q* value = 6.3 × 10^−6^) (Supplementary Table S6).

**Figure 8:**
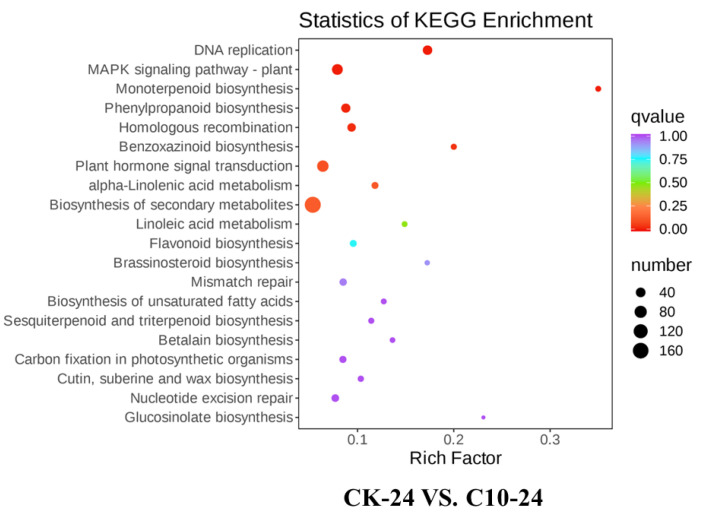

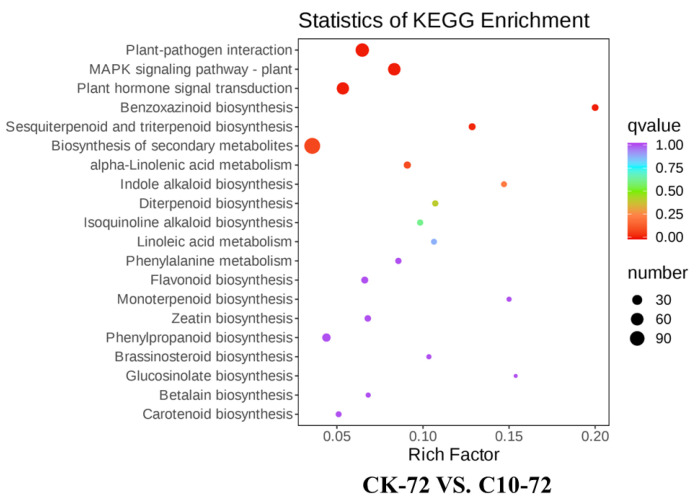
KEGG analysis of DEGs identified using RNA-seq in CK vs. C10 at 24 and 72 h. The size of the black dot represents the gene count. The DEGs were chosen based on a criterion of an adjusted *p* value (*p.adj*) *<* 0.05, as indicated by the colored sidebar.

To better understand the biological function of DEGs in *F. graminearum*-infected maize after COR treatment, an enrichment analysis was performed using the KEGG pathways, and some important enriched KEGG pathways, either upregulated or downregulated, were chosen. KEGG analysis enriched COR responsive DEGs in 8 pathways: DNA replication (KEGG ID: Ko 03030; *q* value = 0), MAPK signaling pathway-plant (KEGG ID: Ko 04016; *q* value = 0.001), monoterpenoid biosynthesis (KEGG ID: Ko 00902; *q* value = 0.002), phenylpropanoid biosynthesis (KEGG ID: Ko 00940; *q* value = 0.009), homologous recombination (KEGG ID: Ko 03440; *q* value = 0.022), benzoxazinoid biosynthesis (KEGG ID: Ko00402; *q* value = 0.032), plant hormone signal transduction (KEGG ID: Ko04075; *q* value = 0.09), and alpha-linolenic acid metabolism (KEGG ID: Ko00592; *q* value = 0.11) in the comparison of CK_24h vs. C10_24h (Supplementary Table S7). Several pathways, including plant–pathogen interaction (KEGG ID: Ko 04626; *q* value = 0), MAPK signaling pathway-plant (KEGG ID: Ko 04016; *q* value =6.9× 10^−11^), plant hormone signal transduction (KEGG ID: Ko 04075; *q* value =9× 10^−6^), benzoxazinoid biosynthesis (KEGG ID: Ko 00402; *q* value = 0.001), sesquiterpenoid and triterpenoid biosynthesis (KEGG ID: Ko00909; *q* value = 0.01), biosynthesis of secondary metabolites (KEGG ID: Ko 01110; *q* value = 0.07), and alpha-linolenic acid metabolism (KEGG ID: Ko00592; *q* value = 0.08) were enriched in the comparison of CK_72h vs. C10_72h (Supplementary Table S8). DEGs were enriched in plant hormone signal transduction, benzoxazinoid biosynthesis, and alpha-linolenic acid metabolism (Figure 8).

Six genes from ko00592: Alpha-linolenic acid metabolism, and ko00360: Phenylalanine metabolism, were selected for qRT-PCR validation of RNA-seq data (Supplementary Figure S3). The expression pattern of genes quantified by qRT-PCR was similar to that of RNA-seq. Thus, the reliability of the RNA-seq used to identify DEGs associated with GRS phenotypes was verified in this study.

### 3.7. Co-Expression Network Analysis for the Candidate Hub Genes Involved in Maize Defense

**Figure 9:**
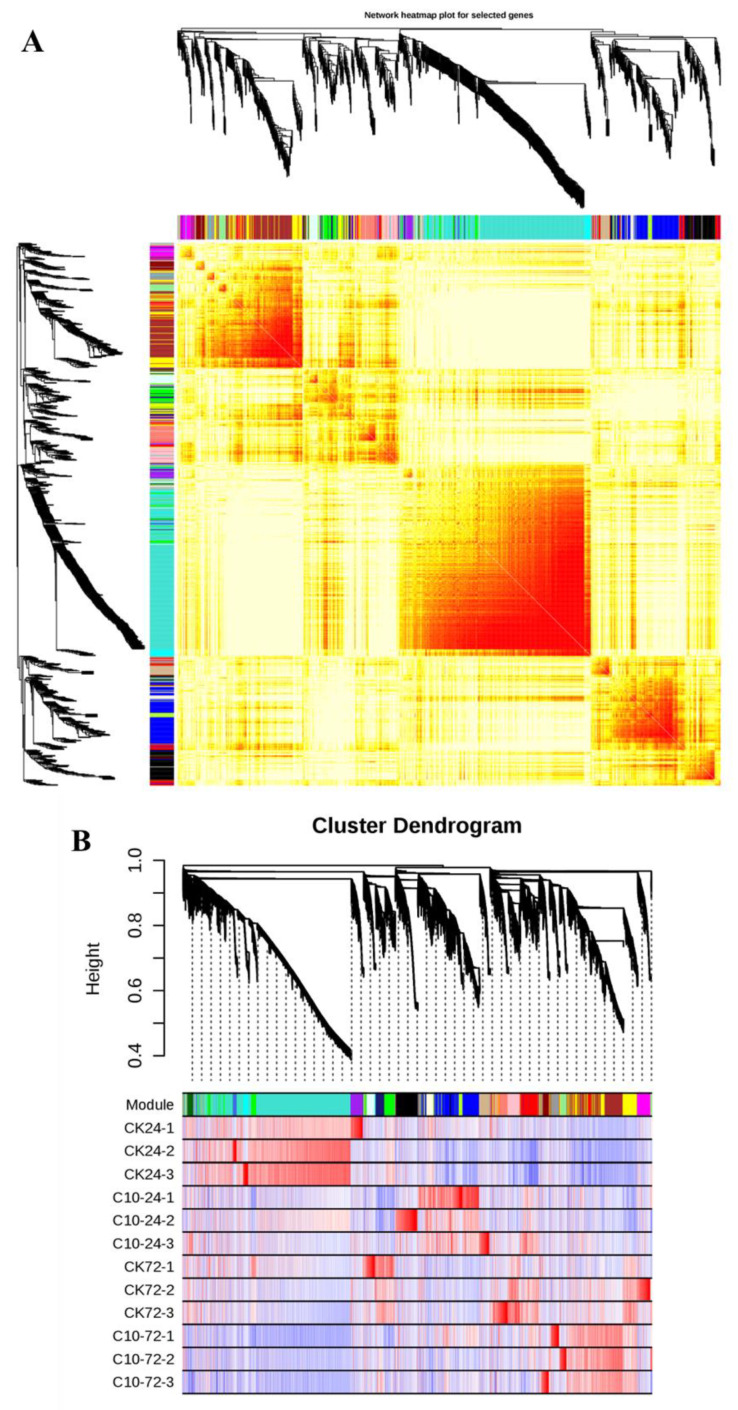

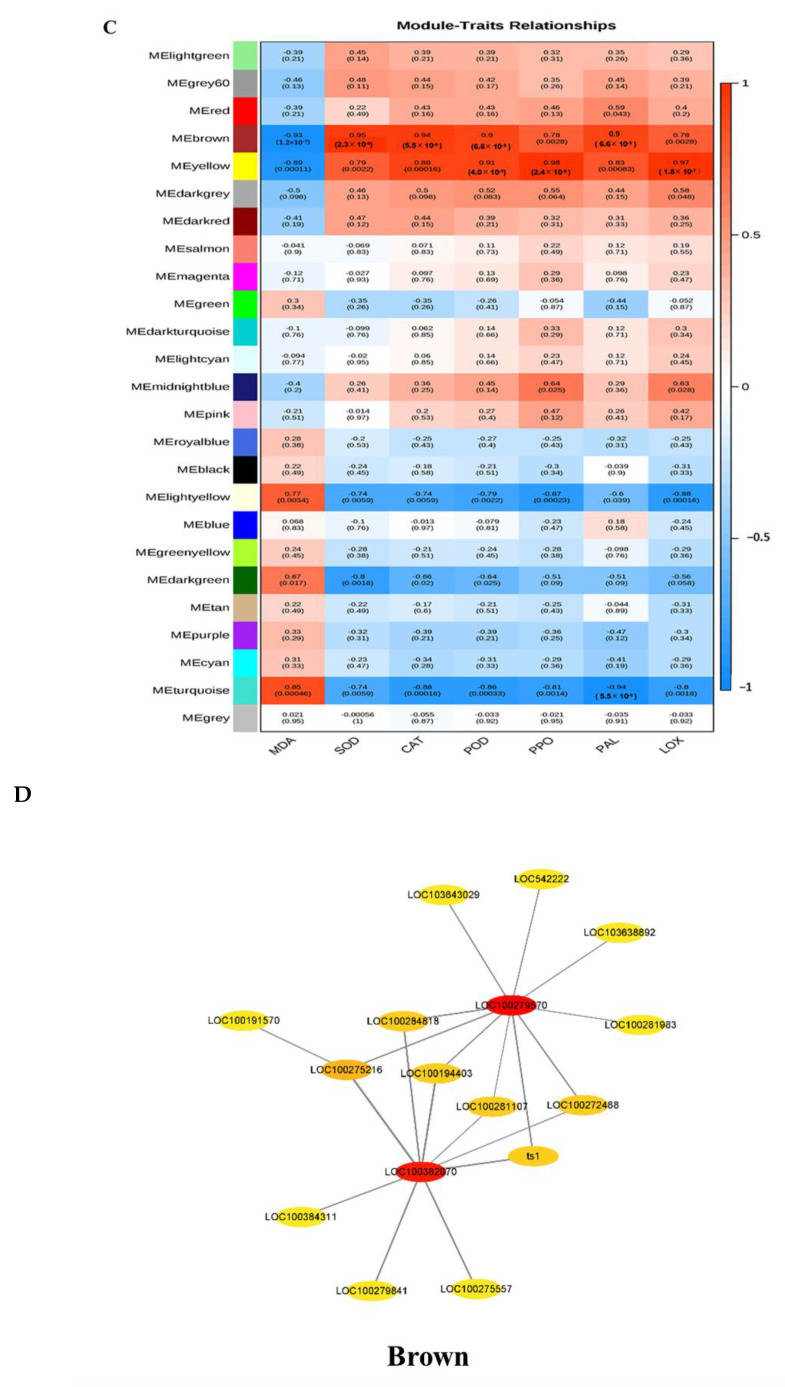

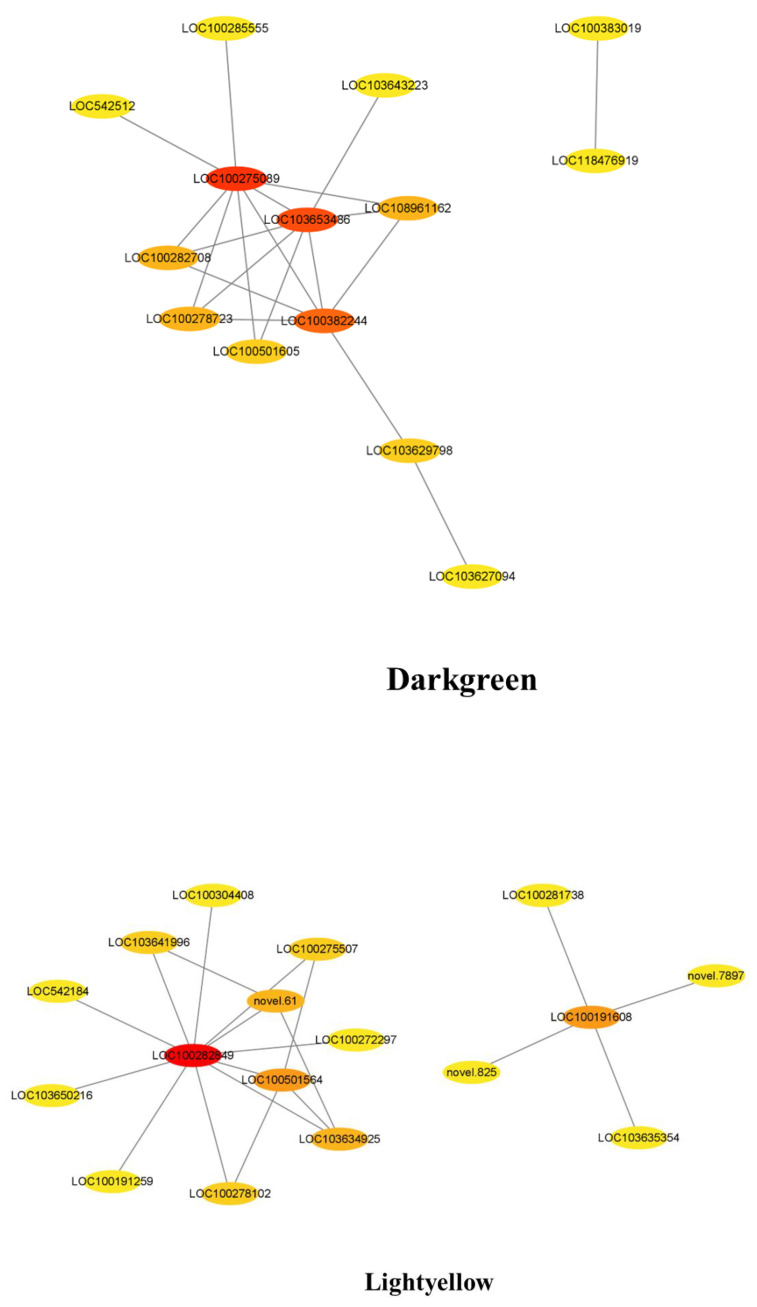

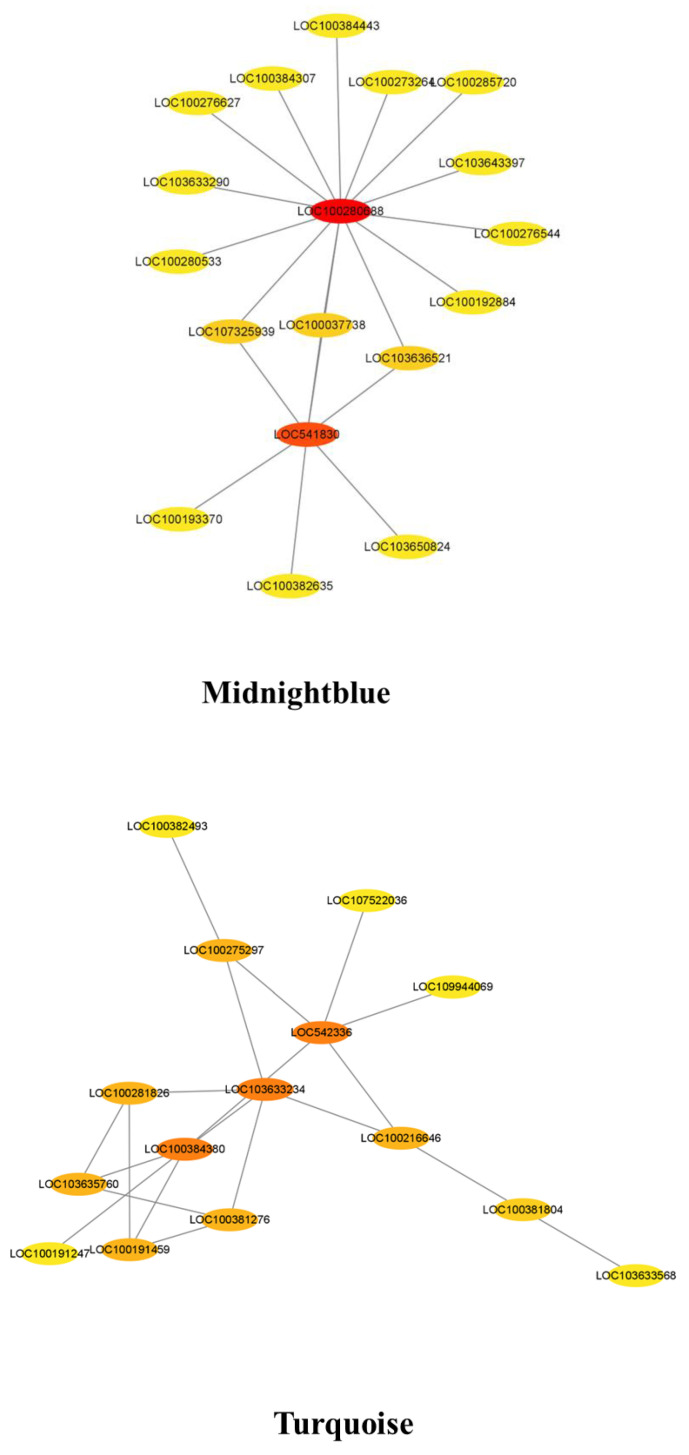

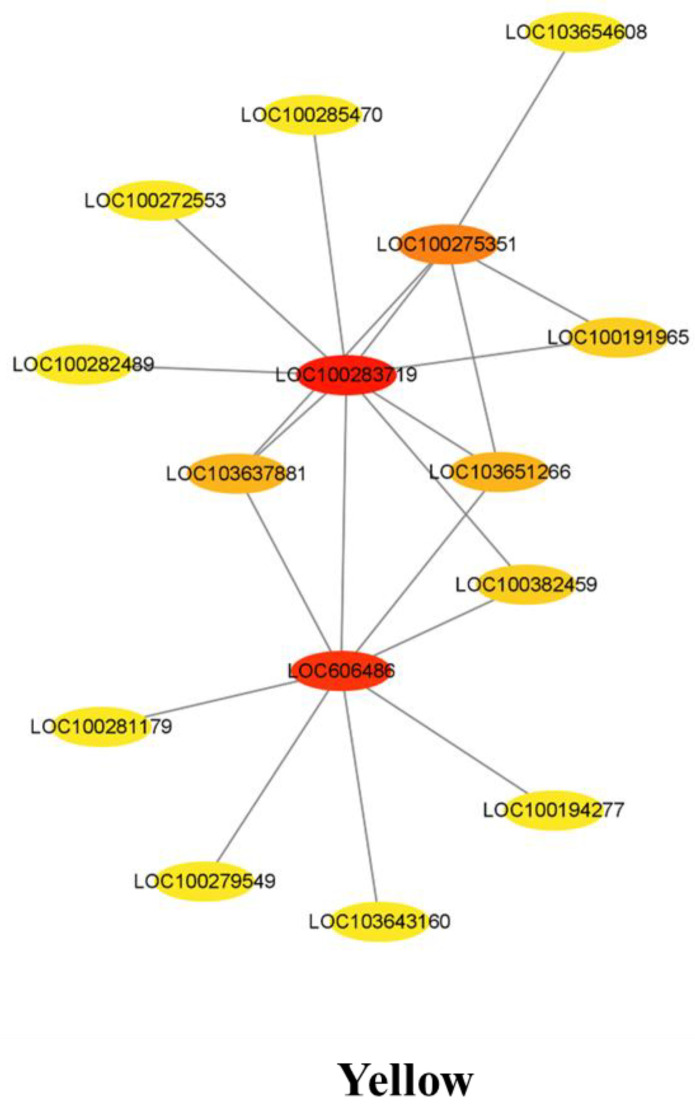
WGCNA to identify key candidate genes involved in maize defense. (**A**) Cluster dendrogram and network heatmap of genes calculated using the co-expression module. (**B**) The hierarchical clustering of 25 modules that share co−expressed genes. Each leaflet on the tree represents a distinct gene. (**C**) Pearson correlations are utilized to determine module–trait relationships. The color key from green to red indicates r^2^ values between −1 and 1. (**D**) Gene networks consist of six modules with substantial correlations to phenotypic attributes. The treatments include untreated control (CK) and 10 g/mL COR (C10).

The FPKM values of 2861 common DEGs and plant biochemical parameters (SOD, POD, CAT, MDA, PPO, PAL, and LOX) at different time points were selected for WGCNA, and correlations between module traits were calculated. Based on co-expression patterns, twenty-five gene modules were identified. Each module was displayed as a cluster dendrogram and network heatmap with a distinct color (Figure 9A–C).

Among the 25 identified modules, only six of the twenty-five identified modules exhibited statistically significant correlations with the phenotypic data. The brown module comprised significant negative correlations with MDA (r^2^ = 0.93) and significant positive correlations with SOD, CAT, POD, PPO, PAL, and LOX with correlation coefficients (r^2^) of 0.95, 0.94, 0.9, 0.78, 0.9, and 0.78, respectively. The yellow module contained significant negative correlations with MDA (r^2^ = 0.89) and significant positive correlations with SOD, CAT, POD, PPO, PAL, and LOX with correlation coefficients (r^2^) of 0.79, 0.88, 0.91, 0.98, 0.83, and 0.97, respectively. The midnight-blue module showed positive correlations with PPO (r^2^ = 0.64) and LOX (r^2^ = 0.63). The light-yellow module comprised significant positive correlations with MDA (r^2^ = 0.77) and significant negative correlations with SOD, CAT, POD, PPO, PAL, and LOX with correlation coefficients (r^2^) of 0.74, 0.74, 0.79, 0.87, 0.6, and 0.88, respectively. The dark-green module showed positive correlations with MDA (r^2^ = 0.67) and negative correlations with SOD (r^2^ = 0.8), CAT (r^2^ = 0.66), and POD (r^2^ = 0.64). The turquoise module comprised significant positive correlations with MDA (r^2^ = 0.85) and significant negative correlations with SOD, CAT, POD, PPO, PAL, and LOX with correlation coefficients (r^2^) of 0.74, 0.88, 0.86, 0.81, 0.94, and 0.8, respectively. The hub genes from these modules were selected to visualize the gene networks using the Cytoscape built-in extension “CytoHubba” (Figure 9D). In addition, gene annotation data was extracted from the maize reference genome (ZmB73_RefGenv4) to identify maize defense-related genes within these gene networks.

### 3.8. COR Enhanced the Expression of Key Candidate Genes in Maize Defense Response to F. graminearum

Two key genes in the brown module were identified: The WRKY transcription factor gene (LOC100279570), which plays an essential role in the regulation of plant defense responses against biotic stress and is involved in plant oxidative stress responses to high ROS levels by reducing the accumulation of H_2_O_2_ and MDA and enhancing the antioxidant enzyme activities; and the calcium-binding protein gene (LOC100382070), which is a negative regulator of plant defense. This decreases COI1-mediated JA sensitivity and the expression of JA-responsive genes, operating as an essential signaling link between Ca^2+^ and JA signaling. Two genes (LOC100275089 and LOC100382244) were identified as key genes in the dark-green module. The NBR1-like protein gene (LOC100275089) is required for the SA signaling pathway to limit excessive senescence and programmed cell death in response to pathogen infection. The amino acid permease gene (LOC100382244), whose primary function is to provide amino acids for protein and enzyme synthesis and the precursors of plant hormones, regulates numerous aspects of plant development, metabolism, and stress response. Similarly, in the midnight-blue module, the glutathione S-transferase gene (LOC541830) was identified as an essential gene for plant oxidative stress response against high ROS levels generated by fungal infection. Two genes (LOC100191608 and LOC100501564) were determined to be essential for the light-yellow module. These genes were identified as HXXXD-type acyl-transferase family protein and protein kinase. The HXXXD-type acyl-transferase family protein gene (LOC100191608) is accountable for the formation of aliphatic and aromatic phenolamides. Its expression depends on JA signaling upon fungal infection to generate systemic signals that translocate to other healthy plant parts and propagate systemic resistance throughout the plant. The prolin-rich extensin-like receptor protein kinase gene (LOC100501564) is involved in phytohormone signaling to initiate a defense response and modulate gene expression in ion homeostasis. In addition, this protein is thought to act as a sensor/receptor to monitor changes in the cell wall during the cell’s expansion. The AP2-like ethylene-responsive transcription factor gene (LOC100384380) participates in the phytohormone signaling pathway and flavonoid biosynthesis pathways to protect the plant and was a key gene in the turquoise module. The basic leucine zipper gene (bZIP, LOC100275351) and glycosyltransferase gene (LOC606486) were identified as key genes in the yellow module. The basic leucine zipper gene (bZIP, LOC100275351) is engaged in signaling and responses to abiotic/biotic stimuli, including osmotic, drought, cold stress, and pathogen defense. In addition, the glycosyltransferase gene (LOC606486) participates in flavonol biosynthesis and is closely related to cellular redox potential and glycosylation-mediated regulation of flavonol accumulation. Table 3 displays the function and expression patterns of all essential maize defense response genes associated with COR.

### 3.9. Metabolite Analysis of Maize in Response to COR

**Figure 10:**
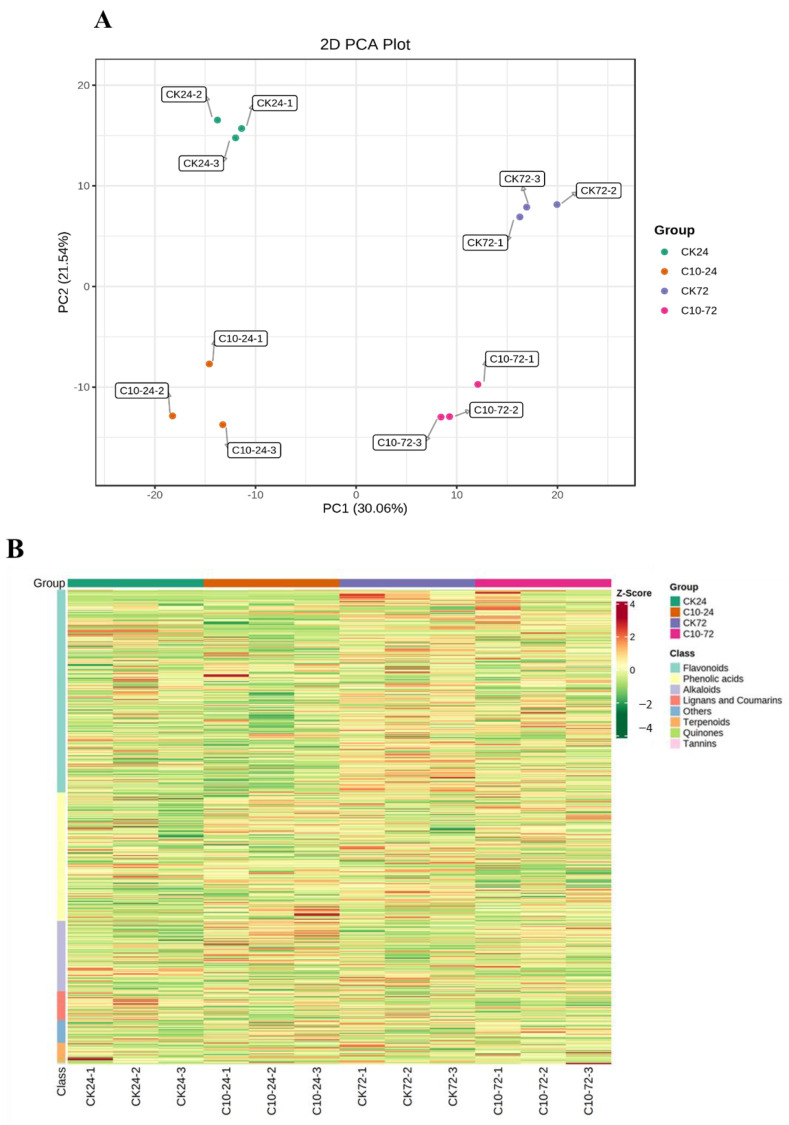

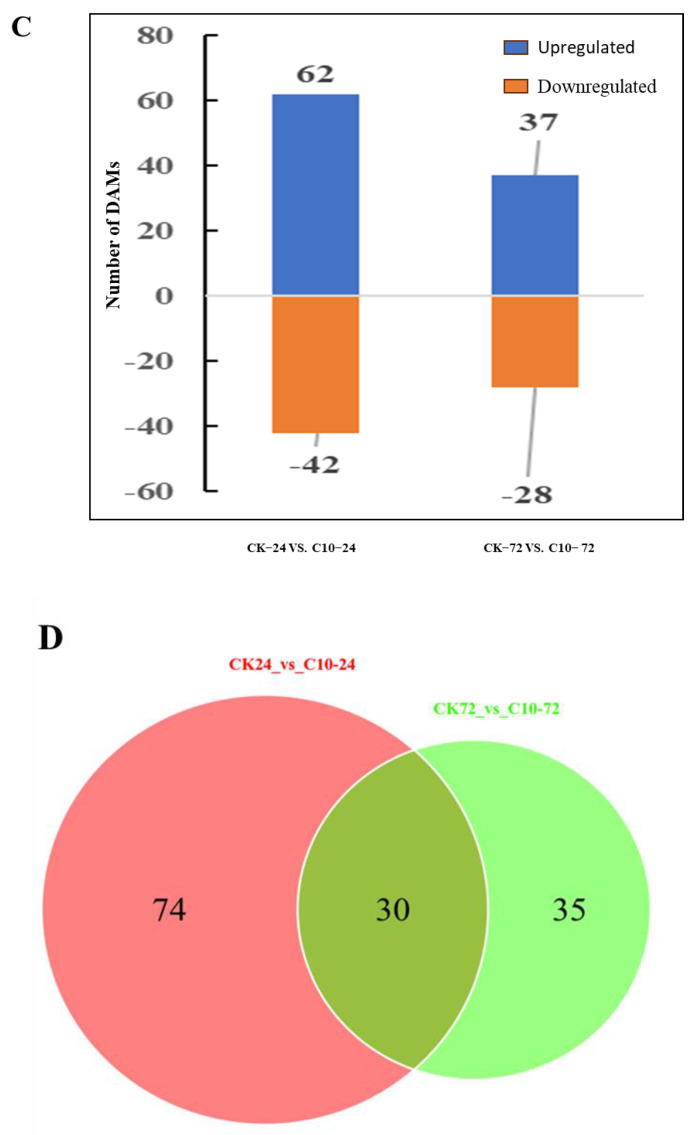

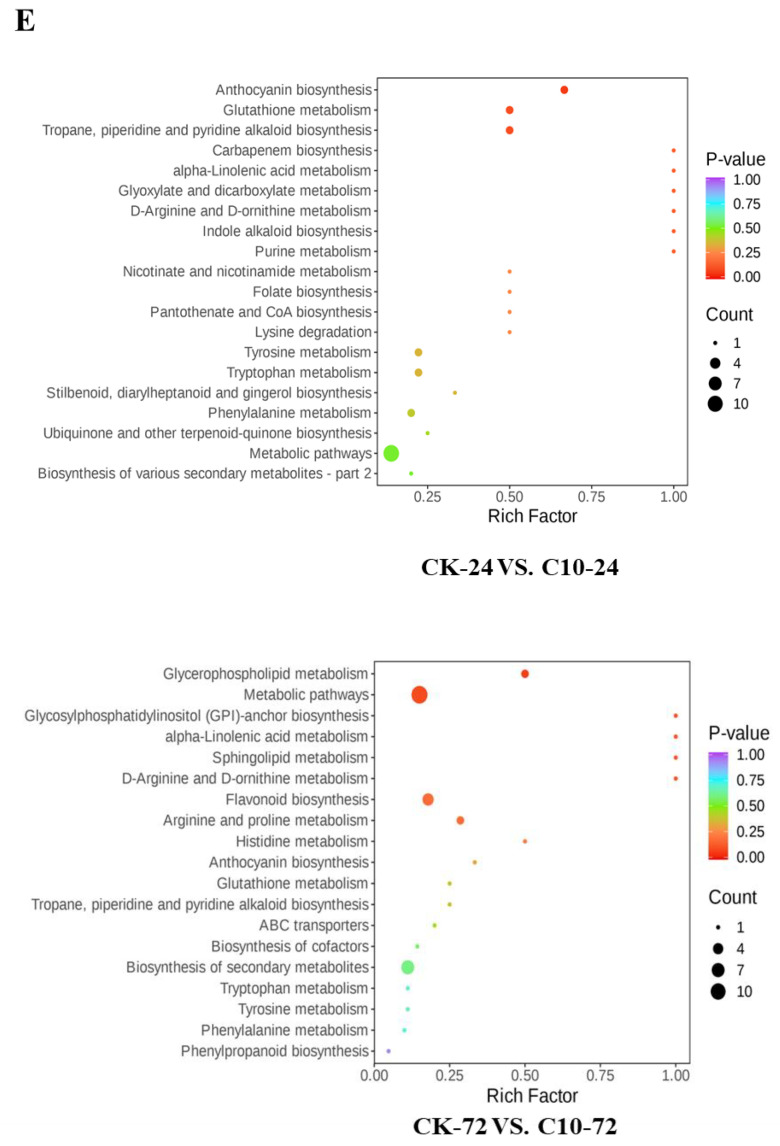
Metabolomic analysis of maize treated with COR at different time points after infection with Fusarium graminearum. (**A**) PCA plot showing divergence in metabolome data among treatments, including the untreated control (CK) and treatment with 10 g/mL COR (C10). (**B**) Hierarchical clustering of abundantly identified metabolites. (**C**) Total number of upregulated and downregulated DAMs identified in CK and C10 at two time points. (**D**) Venn diagram depicting the unique and common DAMs shared by CK and C10. (**E**) The top 20 enriched KEGG terms of the DAMs detected in comparative metabolomics comparing CK and C10 at 24 and 72 h.

To explore the metabolic changes that occur in maize following *F. graminearum* infection by exogenous COR treatment, we analyzed COR-treated maize stem samples at two time points (24 and 72 hpi) using targeted liquid chromatography-tandem mass spectrometry (LC-MS/MS). A total of 742 metabolites were identified and annotated, including 317 flavonoids, 201 phenolic acids, 110 alkaloids, 45 lignans and coumarins, 29 terpenoids, 2 quinonoids, 2 tannins, and 36 other metabolites (Supplementary Table S9). Figure 10A illustrates the PCA clustering of metabolites detected at various time points. The results of the PCA indicated that the first two PCs accounted for 51.6% of the total variation. The plot also showed a clear distinction between the metabolomes of maize plants infected with *F. graminearum* and those of the control plants. The hierarchical clustering heatmap (Figure 10B) indicated that 72 h samples were clustered separately from 24 h samples and control plants. Furthermore, 104 (up: 62; down: 42) and 65 (up: 37; down: 28) DAMs were identified at 24 and 72 hpi compared to CK and C10, respectively (Figure 10C). In addition, a Venn diagram between the two treatments highlighted the number of standard and specific DAMs. At both time points, thirty compounds were substantially affected by COR treatment, and their accumulation is depicted in Figure 10D. Pathways of alpha-linolenic acid metabolism, phenylalanine metabolism, phenylpropanoid, and flavonoid biosynthesis were enriched in the comparison of the two groups, representing fundamental metabolic responses to COR treatment (Figure 10E, Supplemental Table S10).

## 4. Discussion

*Fusarium graminearum* (teleomorph *Gibberella zeae*) is the causal pathogen of *Gibberella* stalk rot in maize and normally overwinters as chlamydospores on crop residue and infects plants by directly penetrating seedling roots or insect or mechanical injury [37]. After pollination, disease symptoms develop rapidly in mild, humid conditions [38]. Despite the fact that *F. graminearum* infection causes significant losses in maize, we have a limited understanding of the contribution of specific defense hormones or functional analogs to maize resistance. The latest research has demonstrated that maize treated with exogenous MeJA results in increased resistance to this pathogen [21]. In this study, we further investigated the effect of COR on the disease phenotype, maize growth, and physiology under artificial inoculation conditions. Furthermore, WGCNA was used to integrate the transcriptome, biochemical data, and untargeted metabolite profiling to identify candidate genes and metabolites associated with the regulation of defensive biochemical enzymes and the induction of maize resistance and defense pathways against *F. graminearum*. 

Plants are frequently challenged by diverse pathogens, necessitating effective strategies to prevent pathogen entry. To optimize the coordination of these defense responses, the plant utilizes a complex array of mechanisms to perceive the presence of the pathogen and then transmit this signal via an interfering set of hormone signal transduction pathways that specifically modulate actual defense outputs [28]. Pathogen-associated molecular pattern (PAMP)-triggered immunity (PTI) and effector-triggered immunity (ETI) are well-described mechanisms of plant immunity against pathogens [39]. PAMPs that bind to pattern recognition receptors allow infected organisms to detect pathogens directly and determine if they are under attack. The infected host can identify pathogens using ETI pattern recognition, thereby alerting the host to pathogens through the associated damage caused by pathogenic toxins or effectors. Immune-related ROS production and MAP kinase activation can occur in PTI and ETI [40]. Numerous previous studies attempted to dissect the intricate molecular mechanisms underlying maize–Fusarium interactions by identifying essential genes using transcriptomic, metabolomic, and functional approaches. Utilizing a high-throughput tandem mass tag (TMT)-based technology for proteomic comparison, Bai et al. (2021) investigated pathogen-responsive proteins and biological processes in maize stems with moderate resistance to *F. graminearum* infection. Several secondary metabolism pathways and defense-related proteins were associated with the defense responses. In addition, it has been established that ZmWRKY83 is a crucial transcription factor for plant disease resistance [41]. Moreover, using integrated gene co-expression analysis and metabolite profiling, it was discovered that ZmHIR3 likely contributes to disease resistance to GSR, probably through the transcriptional regulation of key genes, functional metabolites, and the control of cell death [42].

Because the use of certain agronomic practices and fungicides in soil-borne disease control is either ineffective or detrimental to the environment, significant effort has been devoted to investigating plant immune elicitors. Jasmonates, such as JA and MeJA, can trigger secondary metabolic pathways in plants, resulting in an arsenal of antimicrobial bioactive metabolites (such as isoflavonoids and volatile compounds) with diverse structures and preceding biochemical pathways [43]. Since COR mimics (+)-7-iso-jasmonoyl-L-isoleucine, it has been used as a tool to design synthetic structural analogs that can be used as stimulators of plant defense mechanisms in agriculture [44]. COR reduces the production of ROS by activating antioxidant enzymes and 2,2-diphenyl-1-picrylhydrazyl (DPPH)-radical scavenging, thereby preventing membrane peroxidation and denaturation of biomolecules under salinity stress in cotton [45], and it can enhance cabbage seedlings’ resistance to black rot, which might be related to the variation of antioxidant enzyme activity, the O^2-^generation rate, or MDA content after 1 μg/mL COR application [22]. Our findings are also consistent with the pathogenicity against *F. graminearum*, reducing the severity of the disease and regulating plant growth in COR-treated plants. In response to pathogen infection, plants produce several reactive oxygen species (ROS) scavengers, including SOD, POD, PPO, and PAL. It has been suggested that elicitation is also associated with plant defense responses and the establishment of oxidative stress [35]. To explain the basis of this relationship, we measured the activity of antioxidant and defense enzymes, such as SOD, POD, PAL, and LOX, which sharply increased after 48 hpi. The MDA content visibly declined after 48 hpi. In addition, these enzyme activities were relatively higher in plants treated with 10 μg/mL COR. The results demonstrated that COR altered antioxidative metabolism, which increased the production of protective enzymes to enhance the plant’s defense mechanism.

Stomata are microscopic openings formed by pairs of guard cells in the epidermis of terrestrial plants, which are essential for gas exchange and water loss regulation. Plants regulate stomatal aperture in response to environmental conditions. Stomatal openings are also an important entry point for pathogens into plants, and plants have evolved mechanisms to regulate stomatal apertures as an immune response against pathogen invasion. The accumulation of reactive oxygen species in guard cells is an early hallmark of stomatal movement [46]. Although the stomatal density in the COR-treated and control plants was similar, the length and width of the stomata and aperture in the COR-treated plants were significantly narrower, whereas those of the control plants were broader. The area of the stomata and aperture after COR treatment were also smaller than those in the control after *F. graminearum* infection. Therefore, we also measured H_2_O_2_ accumulation in the leaf cells at different times using an H_2_O_2_ assay kit and observed the fluorescence of the stomatal guard cells with a confocal laser scanning microscope using an H_2_O_2_-selective H_2_DCF-DA staining assay at 24 hpi under normal conditions and COR treatment. These findings corroborate that COR promotes stomatal closure and H_2_O_2_ accumulation in guard cells after *F. graminearum* infection to facilitate GSR resistance.

With rapid developments in bioinformatics, the WGCNA method has been developed to explore the functionality of the transcriptome. The relationship between the trait and the module identifies the most significant hub genes, which is an effective method for investigating the depth mechanism of complex traits. Extensive research conducted over the past several decades has shown that the metabolic network of plants is reprogrammed in response to abiotic and biotic stress to maintain metabolic homeostasis and the synthesis of various defense-related secondary metabolites, such as plant hormones and flavonoids, to mitigate the negative effects of stress. Furthermore, recent research has also shown that phytohormones, phenylpropanoids, lipids, flavonoids, and PAs differentially accumulate and are involved in the plant’s defense response to pathogen infection [47]. We combined transcriptomics and metabolomics to investigate the complex mechanism underlying COR’s regulation of maize GSR resistance.

### 4.1. Effects of COR on Hub Genes in the F. graminearum Associated Molecular Signaling Responses

In plant responses to pathogen infection, early signaling events, such as calcium ion (Ca^2+^) sensors and proteins that regulate the expression of target genes, transcription factors (TFs), and transcription regulators (TRs), play crucial roles. Considering that these signals activate downstream pathways in response to pathogen-mediated stress, observing their induction can illuminate plants’ underlying molecular mechanisms of biotic stress response. Among the hub genes, we identified one calcium-binding protein (LOC100382070) and one WRKY transcription factor (LOC100279570), which showed a high response against *F. graminearum* invasion. CMLs play diverse functions in plant stress perception and development. The transcripts of CML37, CML38, and CML39 are regulated by biotic and abiotic stress, hormone, and chemical treatment [48]. CML24 has been shown to affect abscisic acid (ABA) and ion stress [49]. CML9 modifies plant responses to ABA and abiotic stress [50], whereas CML42 inhibits the JA pathway and aliphatic glucosinolate content to negatively modulate plant defense [51]. WRKYs are implicated in numerous plant hormone signaling pathways. For example, *AtWRKY40* can recognize the W-box regions of abscisic acid (ABA)-induced genes, such as *AtABF4*, *AtABI4*, and *AtABI5*, and suppress their expression [52]. Exogenous ABA induces the expression of the cucumber *CsWRKY46* gene, and by modulating genes in the ABA signaling pathway, *CsWRKY46* overexpression increases cold tolerance in transgenic plants [53]. *ZmWRKY40* overexpression improves drought tolerance in transgenic *Arabidopsis* by modulating stress-related genes, and the ROS content in transgenic lines under drought stress was decreased by enhancing the activities of POD and CAT [54]. These results demonstrate that WRKY TFs serve as regulators in response to hormones and abiotic stress. Glutathione S-transferase (GST, LOC541830) has been identified as a critical gene required for the plant’s oxidative stress response against high ROS levels generated by a fungal infection [55].

### 4.2. Effects of COR on the Activity of Identified Candidate Genes in Phytohormone Signaling and Biosynthesis Pathways

We identified five signaling-related genes, namely the NBR1-like protein gene (LOC100275089), amino acid permease gene (LOC100382244), HXXXD-type acyl-transferase gene (LOC100191608), prolin-rich extensin-like receptor protein gene (LOC100501564), and basic leucine zipper gene (LOC100275351), showing a high response against *F. graminearum* infection after COR treatment. The NBR1-like protein, which is the archetypal selective autophagy receptor, degrades protein aggregates upon heat, oxidative, and drought stress [56], viral capsids [57], and acts in plants’ defense against pathogen infections [58]. Recent research has demonstrated that autophagy modulates NPR1-dependent salicylic acid signaling via a negative feedback loop, which is required to limit excessive senescence in response to pathogen infection [59]. The amino acid permease regulates extensive aspects of plant development, metabolism, and stress resistance. Through amino acid transport, ZmAAP6 may be involved in stress responses mediated by proline, such as temperature stimulus, oxidative stress, and water deprivation responses [60]. The HXXXD-type acyl-transferase family protein synthesizes aliphatic and aromatic phenolamides. Markakis et al. (2012) found that an HXXXD-type acyl-trans-ferase family protein gene (AT2G39980) was one of the top 10 upregulated genes in 3 h ACC-treated *Arabidopsis* roots and was significantly regulated in microarray analyses of the cross-talk between JA and ethylene signaling in *Arabidopsis* seedlings [61]. The prolin-rich extensin-like receptor protein kinase gene (PERK, LOC100501564) participates in the phytohormone signaling pathway to initiate a defense response and to regulate the expression of genes involved in ion homeostasis. In addition, it is believed to act as a sensor or receptor to monitor changes in the cell wall during expansion [62]. PERK4, a proline-rich extensin-like receptor kinase family member, inhibits root growth through intracellular calcium signaling at an early stage of the ABA signaling pathway, as demonstrated by Bai et al. (2009) [63]. The basic leucine zipper gene (bZIP, LOC100275351) is involved in signaling and responses to abiotic/biotic stimuli, including ABA signaling, osmotic, drought, and cold stress, as well as pathogen defense. As in the case of *Ustilago maydis* infection, this result is consistent with the hypothesis that group D proteins may be involved in integrating diverse systemic signals (SA and ethylene) and responses to pathogen attack in *Arabidopsis* [64].

### 4.3. COR Enhances Maize Resistance to GSR by Alpha-Linolenic Acid Metabolism and Flavonoid Biosynthesis

Alpha-linolenic acid can act not only as a strong antioxidant but also as a precursor to the synthesis of JA, which acts as a signaling molecule to stimulate the downstream anti-stress response, including both biotic and abiotic stresses [65,66]. 17-hydroxylinolenic acid is a hydroxylated (on the 17th carbon) version of alpha-linolenic acid produced from alpha-linolenic acid. In this study, 17-hydroxylinolenic acid was found to be significantly elevated in both CK24 vs. C10-24 and CK72 vs. C10-72 in the α-linolenic acid metabolism of maize.

Flavonoids are a class of plant metabolites that can be divided into numerous subclasses based on their chemical structures [67]. They are produced by the sequential catalysis of chalcone synthase (CHS) and chalcone isomerase (CHI), the essential precursors of other flavonoids [68]. Flavonoids are known to contribute to the immune response against various pathogens. For example, the biosynthesis of sakuranetin (a flavanone) in rice has been shown to increase plant resistance to infection by *Fusarium fujikuroi*-caused bakanae [69] and *Magnaporthe oryzae*-caused rice blast [70].

Through metabolomics and transcriptome analysis, we found that the COR treatment of maize infected with *F. graminearum* significantly increased the levels of several flavonoid compounds. We utilized WGCNA to construct co-expression networks in which several genes were found to be significantly enriched in the flavonoid biosynthetic pathway, including the AP2-like ethylene-responsive transcription factor gene (AP2/ERF) and glycosyltransferase. The transcription factor family AP2/ERF regulates a variety of regulatory processes, including plant growth and development, the protection system, metabolism-responsive genes in ethylene signaling pathways, and the biosynthesis pathways of phytohormones in plants [71]. According to Zhao et al., CitCHIL1 is crucial in producing citrus flavanones and flavones by forming a flavonoid metabolon. CitCHIL1 transcriptional regulation is mediated by three AP2/ERF superfamily members. CitRAV1 interacts with CitERF33 to create a transcription complex that stimulates CitCHIL1 expression and flavonoid production [72].

Glycosylation is a common modification reaction and is frequently the last stage in the biosynthesis of flavonoids [73]. Flavonoids predominantly exist as their glycosides, making them more stable and soluble, facilitating their transport and accumulation within plant cells [74]. Recent research indicates that two flavonoid UGTs, UGT79B2 and UGT79B3, increase plant tolerance to abiotic stress by regulating anthocyanin accumulation and eliminating ROS [75]. In addition, UFGT2 overexpression reduces H_2_O_2_ production in plants. These findings suggest that a close relationship between cellular redox potential and flavonol glycosylation regulates flavonol accumulation [76]. Future research will focus on the differential regulation of genes implicated in oxidative stress response and flavonoid biosynthesis.

## 5. Conclusions

The present study, by comprehensive physiological, biochemical, transcriptomic, metabolomic, and phytohormone analysis in COR-treated maize inoculated with *F. graminearum*, indicated dynamic transcriptional and metabolite changes in maize stems after COR application. COR treatment strongly enhanced disease resistance and promoted stomatal closure with H_2_O_2_ accumulation, and 10 μg/mL was confirmed as the best concentration. Meanwhile, COR treatment increased defense-related enzyme activity and decreased the malondialdehyde content with enhanced antioxidant enzyme activity. Moreover, we integrated transcriptomic and metabolomic data to systemically explore the defense mechanisms of COR, and multiple hub genes were pinpointed using weighted gene correlation network analysis (WGCNA). These genes are highly correlated with the jasmonic acid–ethylene signaling pathway and antioxidants. Interestingly, some differential accumulation of metabolites (DAMs) were related to alpha-linolenic acid metabolism and flavonoid biosynthesis, which may be one of the key pathways underpinning maize responses to COR treatment. Future research should concentrate on the molecular mechanisms and roles underlying early signaling events and transcription factors in this process. Overall, this research not only elucidated the regulatory mechanism underpinning GSR stress resistance in response to COR treatment but also provided a sustainable and environmentally friendly strategy for controlling the maize GSR disease.

## Figures and Tables

**Table 1:** Differential effect of COR on maize growth under *Fusarium graminearum* infection.

Treatments	Plant Height (cm)	Stem Diameter (cm)	Leaf Length (cm)	Leaf Width (cm)	SPAD	Fresh Weight (g)	Dry Weight (g)
CK	59.80 ± 6.42 ^a^	11.02 ± 1.20 ^a^	51.20 ± 2.29 ^b^	4.24 ± 0.16 ^a^	23.26 ± 1.11 ^a^	29.21 ± 3.05 ^a^	2.72 ± 0.36 ^a^
COR1	56.90 ± 6.11 ^a^	9.40 ± 1.25 ^b^	60.90 ± 3.96 ^a^	3.60 ± 0.37 ^b^	22.60 ± 2.70 ^a^	27.30 ± 4.54 ^a^	2.10 ± 0.37 ^ab^
COR10	50.13 ± 6.21 ^b^	9.45 ± 1.12 ^b^	45.93 ± 2.72 ^c^	3.91 ± 0.55 ^ab^	21.43 ± 2.45 ^a^	27.56 ± 3.35 ^a^	2.19 ± 0.36 ^ab^
COR50	43.82 ± 3.39 ^b^	9.39 ± 1.28 ^b^	45.06 ± 2.64 ^c^	3.57 ± 0.46 ^b^	20.57 ± 3.17 ^a^	20.48 ± 4.13 ^b^	1.92 ± 0.57 ^b^

Different lowercase letters following mean ± SD represent significant differences between the treatments (*p* < 0.05). Treatments include the administration of 1 μg/mL COR (COR1), 10 μg/mL COR (COR10) and 50 μg/mL COR (COR50).

**Table 2:** Effects of COR treatment on stomatal traits.

Treatments	Stomatal Length (um)	Stomatal Width (um)	Aperture Length (um)	Aperture Width (um)	Stomatal Density (Number/mm^2^)	Stomatal Area (um^2^)	Aperture Area (um^2^)
CK	51.59 ± 5.46 ^a^	25.92 ± 0.95 ^a^	51.13 ± 3.09 ^a^	8.51 ± 0.92 ^a^	67.20 ± 5.19 ^a^	1075.31 ± 51.36 ^a^	326.54 ± 17.73 ^a^
COR10	43.58 ± 1.67 ^b^	21.05 ± 3.60 ^b^	34.74 ± 1.7 ^b^	7.79 ± 0.66 ^b^	65.28 ± 3.94 ^a^	904.16 ± 34.45 ^b^	217.93 ± 17.36 ^b^

Different lowercase letters following mean ± SD represent significant differences between the treatments (*p* < 0.05). Treatments include (CK) untreated control and (COR10) treatment with 10 μg/mL COR.

**Table 3:** Selected hub genes from all co-expressed modules with functional annotation.

Module	Gene ID	Annotation	Identified as Key Candidate Gene
Black	LOC103641718	Uncharacterized	×
Blue	LOC100382049	K(+) efflux antiporter 3 chloroplastic	×
Brown	LOC100279570	WRKY transcription factor 40	√
Brown	LOC100382070	Calcium-binding protein CML42 Calcium-binding protein	√
Cyan	LOC100127513	ZCN2 protein	×
Dark green	LOC100275089	NBR1-like protein	√
Dark green	LOC100382244	Amino acid permease 6	√
Dark grey	LOC100274778	Uncharacterized	×
Dark red	LOC100127520	MFT2-Corn MFT-like protein	×
Dark turquoise	LOC118473652	Photosystem I P700 chlorophyll a apoprotein A2	×
Green	LOC103625941	Receptor-like kinase TMK2	×
Green yellow	LOC100272716	Tryptophan aminotransferase	×
Grey60	LOC100273423	Phospholipase A1-IIgamma	×
Light cyan	LOC109945353	Uncharacterized	×
Light green	BA1	bHLH transcription factor	×
Light yellow	LOC100282849	Alanine aminotransferase 2	×
Light yellow	LOC100191608	HXXXD-type acyl-transferase family protein	√
Light yellow	LOC100501564	Prolin-rich extensin-like receptor protein kinase	√
Magenta	novel.8180	Uncharacterized	×
Midnight blue	LOC100280688	D-3-phosphoglycerate dehydrogenase 2 chloroplastic	×
Midnight blue	LOC541830	Glutathione S-transferase	√
Pink	LOC100273965	Major facilitator superfamily defense 1	×
Purple	LOC100127521	ZCN10 protein	×
Red	LOC100279184	TOM1-like protein 9	×
Royal blue	LOC100136884	PTO-like protein kinase	×
Salmon	LOC100193868	Uncharacterized	×
Tan	LOC100193845	Pollen proteins Ole e I like	×
Turquoise	LOC100384380	AP2-like ethylene-responsive transcription factor	√
Turquoise	LOC542336	Cyclin 2	×
Turquoise	LOC103633234	Abnormal spindle-like microcephaly-associated protein homolog	×
Yellow	LOC100283719	Uncharacterized	×
Yellow	LOC606486	Glycosyltransferase	√
Yellow	LOC100275351	Basic leucine zipper 25	√

×, Not key candidate gene. √, Key Candidate Gene.

## Data Availability

The data presented in this study are available on request from the corresponding author upon reasonable request.

## Supplementary Materials

The following supporting information can be downloaded at: https://www.mdpi.com/article/10.3390/jof9121155/s1, Supplementary Figure S1. Hyphae micrographs in *Fusarium graminearum* inoculated maize stems. Supplementary Figure S2. Top enriched resistance-related GO pathways in CK vs. C10 at 24 and 72 h. Supplementary Figure S3. qRT-PCR validation of six DEGs, including the alpha-linolenic acid metabolism pathway genes (A) and phenylalanine metabolism pathway genes (B). Supplementary Table S1. Primers used in the present qRT-PCR validation. Supplementary Table S2. The comparison efficiency of clean reads aligned with a designated reference genome (ZmB73_RefGen_v4). Supplementary Table S3. All up- and down-regulated DEGs of 10 μg/mL COR treatment with maize in response to *Fusarium graminearum* infection at 24 h. Supplementary Table S4. All up- and down-regulated DEGs of 10 μg/mL COR treatment with maize in response to *Fusarium graminearum* infection at 72 h. Supplementary Table S5. GO pathway enrichment analysis for DEGs with CK_24hvs.C10_24h. Supplementary Table S6. GO pathway enrichment analysis for DEGs with CK_72hvs.C10_72h. Supplementary Table S7. KEGG pathway enrichment analysis for DEGs with CK_24hvs.C10_24h. Supplementary Table S8. KEGG pathway enrichment analysis for DEGs with CK_72hvs.C10_72h. Supplementary Table S9. All DAMs of 10 μg/mL COR treatment with maize in response to *F. graminearum* infection at 24 and 72 h. Supplementary Table S10. KEGG pathway enrichment analysis for DAMs with CK_24hvs.C10_24h and CK_72hvs.C10_72h.
