# Supplementary figures and images for: Coronatine-Induced Maize Defense against Gibberella Stalk Rot by Activating Antioxidants and Phytohormone Signaling
Source: J Fungi (Basel). 2023 Nov 30;9(12):1155. doi: 10.3390/jof9121155 (PMC10744721; doi:10.3390/jof9121155)

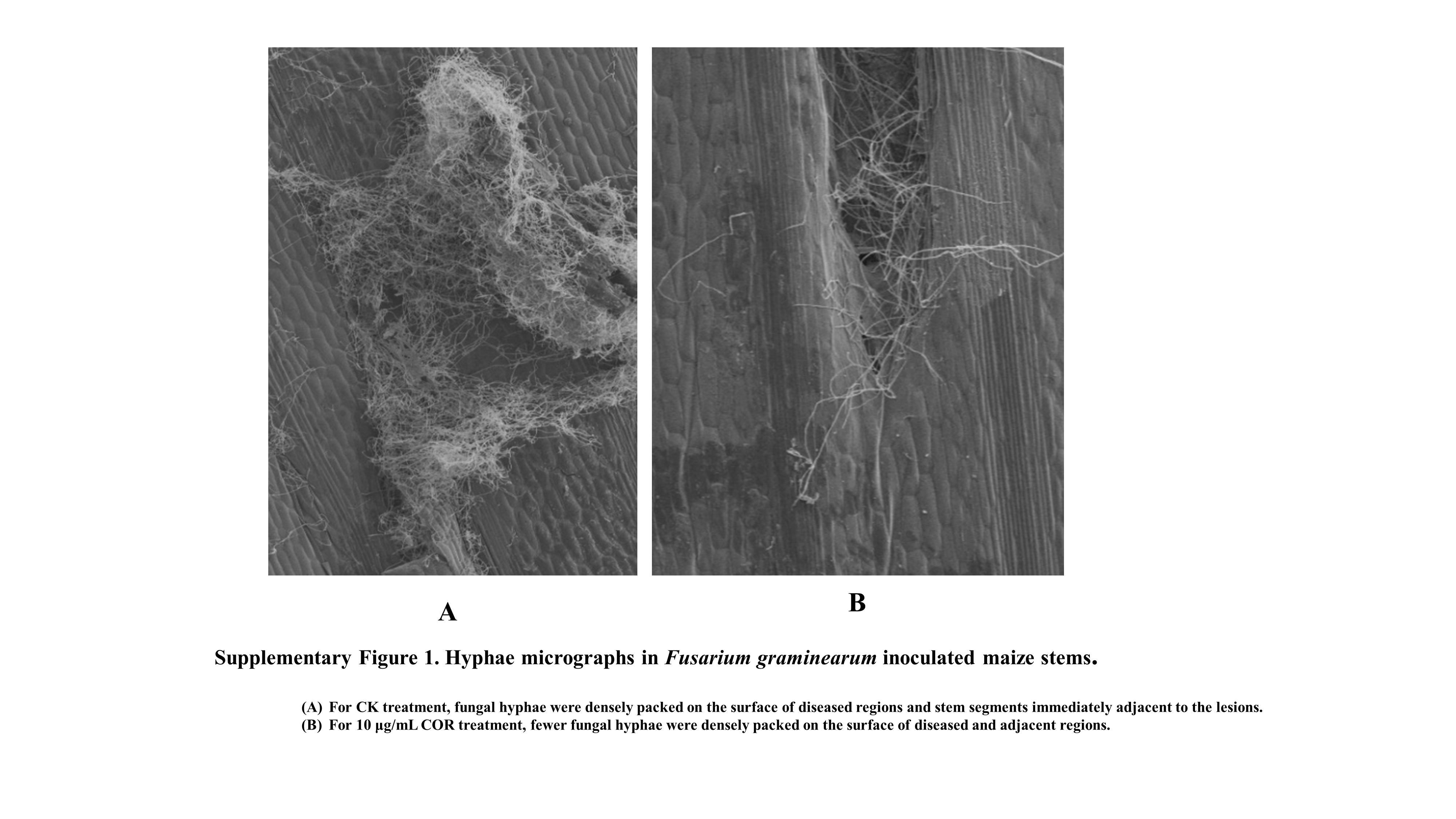

Supplement: Supplementary file 1 [file jof-09-01155-s001.zip › Supplementary Figure S1.tif]

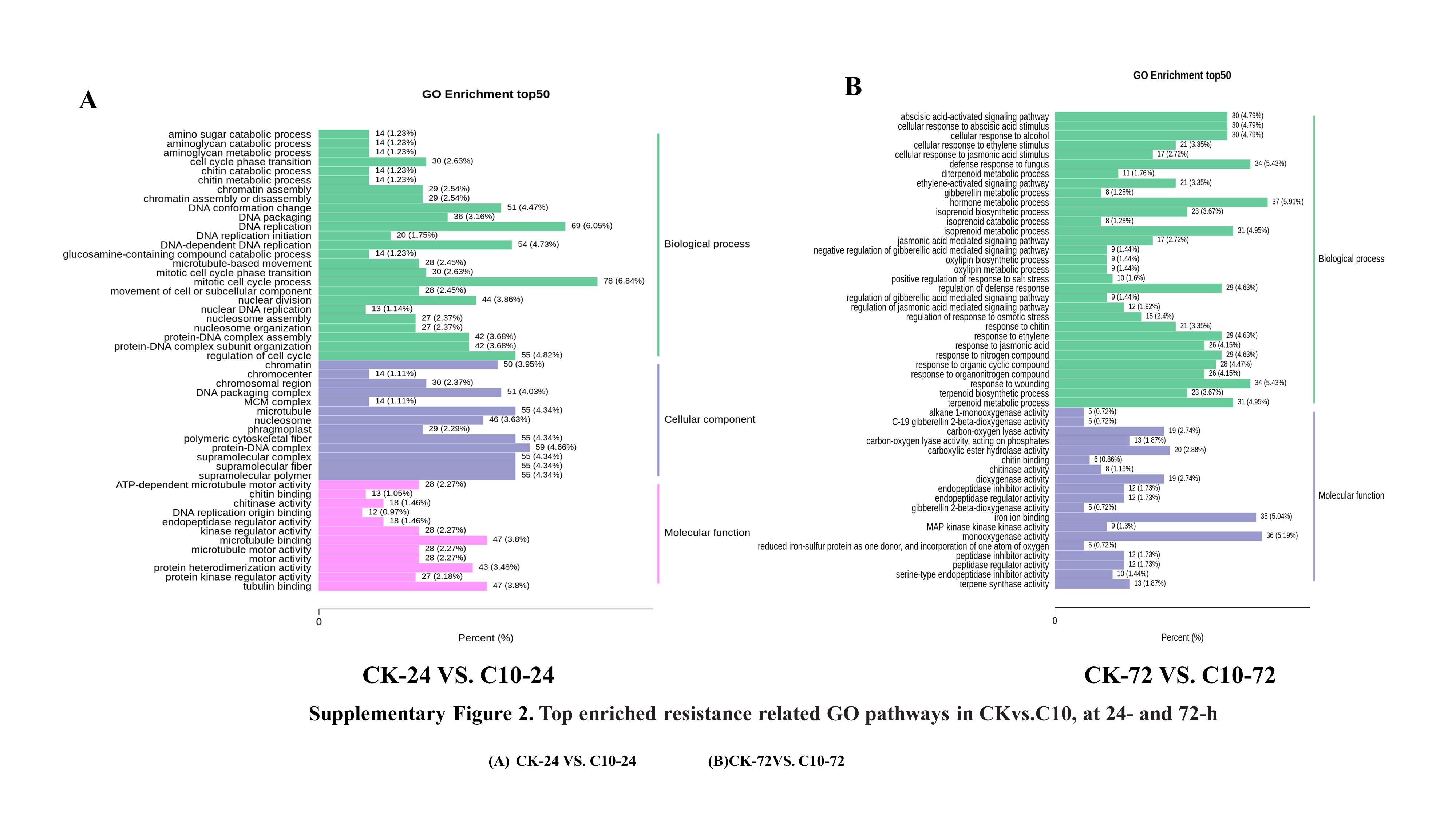

Supplement: Supplementary file 1 [file jof-09-01155-s001.zip › Supplementary Figure S2.tif]

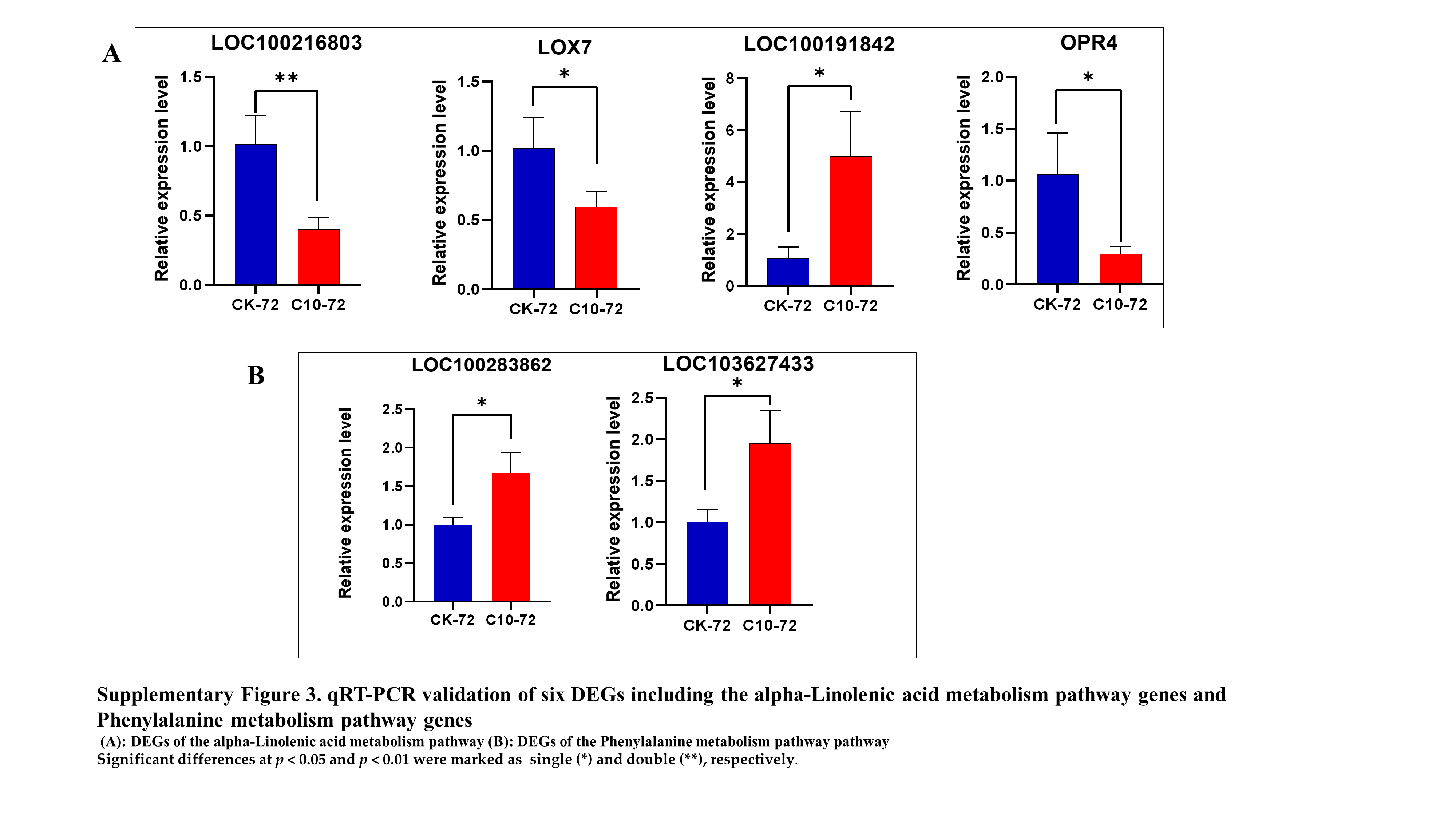

Supplement: Supplementary file 1 [file jof-09-01155-s001.zip › Supplementary Figure S3.tif]
